# CXCL12, a potential modulator of tumor immune microenvironment (TIME) of bladder cancer: From a comprehensive analysis of TCGA database

**DOI:** 10.3389/fonc.2022.1031706

**Published:** 2022-11-07

**Authors:** Jinyan Wang, Yizhao Xie, Dongmei Qin, Shanliang Zhong, Xichun Hu

**Affiliations:** ^1^ Department of Oncology, Fudan University Shanghai Cancer Center, Shanghai, China; ^2^ Department of Oncology, Shanghai Medical College, Fudan University, Shanghai, China; ^3^ Department of Pathology, Nanjing Jiangning Hospital, The Affiliated Jiangning Hospital of Nanjing Medical University, Nanjing, China; ^4^ Center of Clinical Laboratory Science, The Affiliated Cancer Hospital of Nanjing Medical University & Jiangsu Cancer Hospital & Jiangsu Institute of Cancer Research, Nanjing, China

**Keywords:** bladder cancer, tumor immune microenvironment (TIME), tumor-infiltrating immune cells (TICs), CXCL12, biomarker

## Abstract

**Background:**

Tumor immune microenvironment (TIME) plays a significant role in the initiation and progression of bladder urothelial carcinoma (BLCA). However, there are only a few researches regarding the association between immune-related genes and tumor-infiltrating immune cells (TICs) in TIME of BLCA.

**Methods:**

We calculated the proportion of immune/stromal component and TICs of 414 BLCA samples and 19 normal samples downloaded from TCGA database with the help of ESTIMATE and CIBERSORT algorithms. Differentially expressed genes (DEGs) were obtained from the comparison between Stromal and Immune Score and further analyzed by GO and KEGG enrichment analysis, as well as PPI network and COX regression analysis. CXCL12 was overlapping among the above analyses. Single gene analysis of CXCL12 was carried out through difference analysis, paired analysis and GSEA. The association between CXCL12 and TICs was assessed by difference analysis and correlation analysis.

**Results:**

Immune and stromal component in TIME of BLCA were associated with patients’ clinicopathological characteristics. 284 DEGs were primarily enriched in immune-associated activities, among which CXCL12 was the most significant gene sharing the leading nodes in PPI network and being closely related with patients’ survival. Single gene analysis and immunohistochemistry revealed that CXCL12 was down-regulated in BLCA samples and significantly related with the clinicopathological characteristics of patients. Further analysis suggested that CXCL12 was involved in the immune-associated activities probably through its close cross-talk with TICs.

**Conclusions:**

CXCL12 down-regulation could be a potential biomarker to predict the unbalanced immune status of TIME of BLCA, which might provide an extra insight for the immunotherapy of BLCA.

## Introduction

Since the application of cisplatin-based chemotherapy in the mid-1980s, only a few advances have been made in the treatment of bladder urothelial carcinoma (BLCA) (1). Fortunately, immunotherapy has emerged as a novel potential therapy recently. Several clinical trials, such as IMvigor 210 study (2) and CheckMate 275 study (3), revealed that some BLCA patients benefited greatly from the treatment of immune-checkpoint blockade (ICB). Although multiple biomarkers have been associated with the prediction of immunotherapy effect, including the expression of programmed cell death protein 1 (PD-1), PD-L1 and tumor mutation burden (TMB), it is still less than satisfactory to select BLCA patients who are likely to benefit most from immunotherapy (4).

In recent years, the tumor microenvironment (TME) has drawn our attention as its important role in modulating the initiation and progression of cancers, including BLCA (5–10). TME is composed of nonmalignant cells, vessels, lymphoid organs or lymph nodes, nerves, intercellular components and metabolites (11). In brief, stromal component and immune component constitute the TME (12). Furthermore, accumulating research found that tumor immune microenvironment (TIME) had great potential in influencing tumor initiation, predicting immunotherapeutic responsiveness and new therapeutic targets (13). Numerous studies manifested that immune-related genes (IRGs), which were obtained from TME, could predict the survival of cancer patients, including breast cancer (14), endometrial cancer (15), liver cancer (16), gastric cancer (5), bladder cancer (17) and so on. For example, Q. Ding, et al. (18) found that a nine-gene signature was closely related with immune infiltration in TME and the survival of ovarian cancer patients. Besides, tumor-infiltrating immune cells (TICs) in TIME, such as tumor-infiltrating lymphocytes (TILs) and tumor-associated macrophages (TAMs), had the potential to be biomarkers and predictors of multiple cancers (19–21). For instance, elevated level of TILs was associated with better overall survival (OS), higher pathological complete response (pCR) rate, lower risk of recurrence, and more benefit from trastuzumab treatment in breast cancer (22–25). Deficient CD4+ T cells helped to suppress the response of cytotoxic T lymphocytes (CTLs), which means they could establish efficient and durable antitumor activity (26). TAMs acted to inhibit T cell recruitment and modulate the immunity of various tumors, thus affecting the response of immunotherapy (27). Galectin-9+ TAMs predicted prognosis and response to adjuvant chemotherapy in BLCA patients (28). However, there are few researches regarding the association between IRGs and TICs in the TME of BLCA. The exploration of the relationship between IRGs and TICs in TIME could provide us a new sight into the progression and immunotherapy of BLCA.

Fortunately, with the rapid development of transcriptome profiling based on functional genomics analysis, comprehensive analysis of IRGs and TICs in the TIME of BLCA has become possible. In our study, we applied ESTIMATE and CIBERSORT algorithms to calculate the proportion of immune and stromal component, as well as TICs proportion of BLCA samples from The Cancer Genome Atlas (TCGA) database. Next, we started with differentially expressed genes (DEGs) acquired from the comparison of immune and stromal component in BLCA samples, and found out that CXC chemokine ligand-12 (CXCL12) acted to be a potential biomarker and a promising modulator of TIME through its communicating with multiple TICs,

## Methods

### Data acquisition

The transcriptome profiling and clinical data of 414 BLCA samples and 19 normal samples were downloaded from TCGA database (https://portal.gdc.cancer.gov/).

### The assessment of immune component, stromal component and the total component in the TME of BLCA

We applied ESTIMATE algorithm to calculate the proportion of immune and stromal component of each tumor sample in R language (version 3.6.3). Immune Score represented the ratio of immune component in TME of tumor samples. Stromal Score represented the ratio of stromal component in TME of tumor samples. ESTIMATE Score represented the sum of immune and stromal component in TME of tumor samples. The higher Score indicated the larger proportion of the corresponding immune or stromal component in TME.

### Difference analysis between scores and the clinicopathological characteristics

Difference analysis was conducted to learn the correlation between Immune/Stromal/ESTIMATE Score and clinicopathological characteristics, such as age, gender, pathology grade, stage and TNM classification. It was also carried out to find the association between the expression of CXCL12 and clinicopathological characteristics as well as the association between the expression of CXCL12 and TICs. Wilcoxon test was used to compare two groups, and Kruskal-Wallis followed by *post-hoc* Dunn test was used for multiple groups. P < 0.05 was considered to be statistically significant.

### DEGs acquisition

414 BLCA samples were grouped into to two subgroups, including high Immune/Stromal Score group and low Immune/Stromal Score group based on the comparison with the median. Package limma in R was applied for the analysis. A fold change (FC, log_2_^(high score/low score)^) > 2 and false discovery rate (FDR) <0.05 were used to search the DEGs. Pheatmap package in R was used to plot the heatmaps of DEGs.

### Intersection analysis

VennDiagram package in R was used to plot the venn diagram of DEGs.

### Gene ontology and kyoto encyclopedia of genes and genomes enrichment analysis

GO and KEGG enrichment analysis of the above DEGs were further carried out by using the ClusterProfiler, enrichplot, and ggplot2 packages in R. P< 0.05 was considered to be statistically significant.

### Protein–protein interaction network and gene set enrichment analsis

The preliminary PPI network of 284 DEGs was acquired from the Search Tool for Retrieval of Interacting Genes/Proteins (STRING) database (version 11.0) and further reconstructed in Cytoscape (3.6.1). The confidence of interactive relationship of the nodes was >0.95. GSEA (4.1.0) based on the different gene sets, was applied to learn the specific functional profile of CXCL12. P< 0.05 was considered to be statistically significant.

### COX regression analysis

Package survival in R was applied to conduct univariable COX regression.

### Difference analysis and paired analysis of CXCL12

Package Beeswarm in R was used to assess the expression of CXCL12 in bladder tumor samples and normal samples. Packages Ggpubr and BiocManager were applied to learn the expression of CXCL12 in bladder tumor samples and the paired normal samples. Wilcoxon test was used for the comparison. P < 0.05 was considered to be statistically significant.

### Survival analysis

Packages survival and survminer in R were used to carry out the survival analysis. Kaplan–Meier plot and log-rank tests were conducted to learn the associations between the expression of CXCL12 and the survival of BLCA patients. P < 0.05 was considered to be statistically significant.

### TICs Profile

TICs profile in BLCA samples was evaluated by CIBERSORT algorithm.

### Correlation analysis

Correlation analysis was carried out by using spearman’s correlation analysis.

### Tissue samples acquisition and Immunohistochemistry

BLCA and corresponding normal tissues were harvested from BLCA patients at Nanjing Jiangning Hospital. The study was permitted by the Ethics Committee of Nanjing Jiangning Hospital. Slides (4μm) of formalin-fixed paraffin-embedded tissue sections were incubated with CCL19 (1:200; Proteintech) antibody. The specific procedure was consistant with that described in our previous study (29).

## Results

### Analysis process of the study

**Figure 1:**
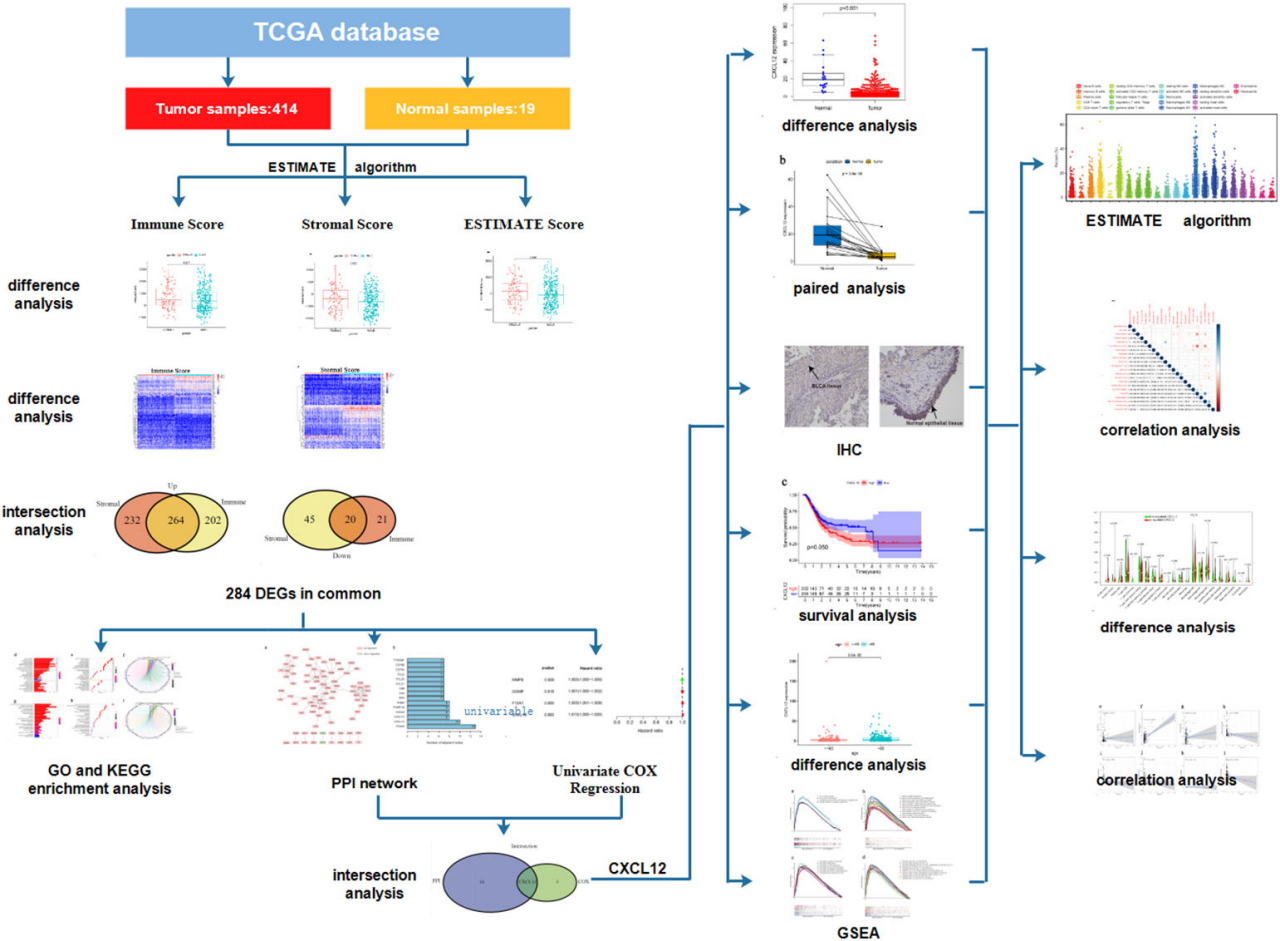
The analysis process of the study.

We downloaded the transcriptome profiling and clinical data of 414 BLCA samples and 19 normal samples from TCGA database and further analyzed the proportion of immune and stromal component in TME of each tumor sample through ESTIMATE algorithm. Difference analysis was conducted to find out the association between immune/stromal component and the clinicopathological characteristics of BLCA patients. A total of 284 DEGs were further acquired based on the immune and stromal component in TME of BLCA. GO and KEGG enrichment analysis of these 284 DEGs were performed to learn their biological functions. Finally, CXCL12 was found out through the intersection analysis of PPI network and univariable COX regression. Then, we focused on the expression of CXCL12 in BLCA samples and normal samples, the association between the expression of CXCL12 and survival and clinic–pathological characteristics of BLCA patients. GSEA of different gene collections was also carried out to learn the function of CXCL12. TICs profile in TIME of BLCA samples was calculated by using CIBERSORT algorithm. Difference analysis and correlation analysis were applied to find out the correlation between the expression of CXCL12 and TICs. The analysis process was shown in **Figure 1**.

### Immune and stromal component in TME were associated with the clinicopathological characteristics of BLCA

**Figure 2:**
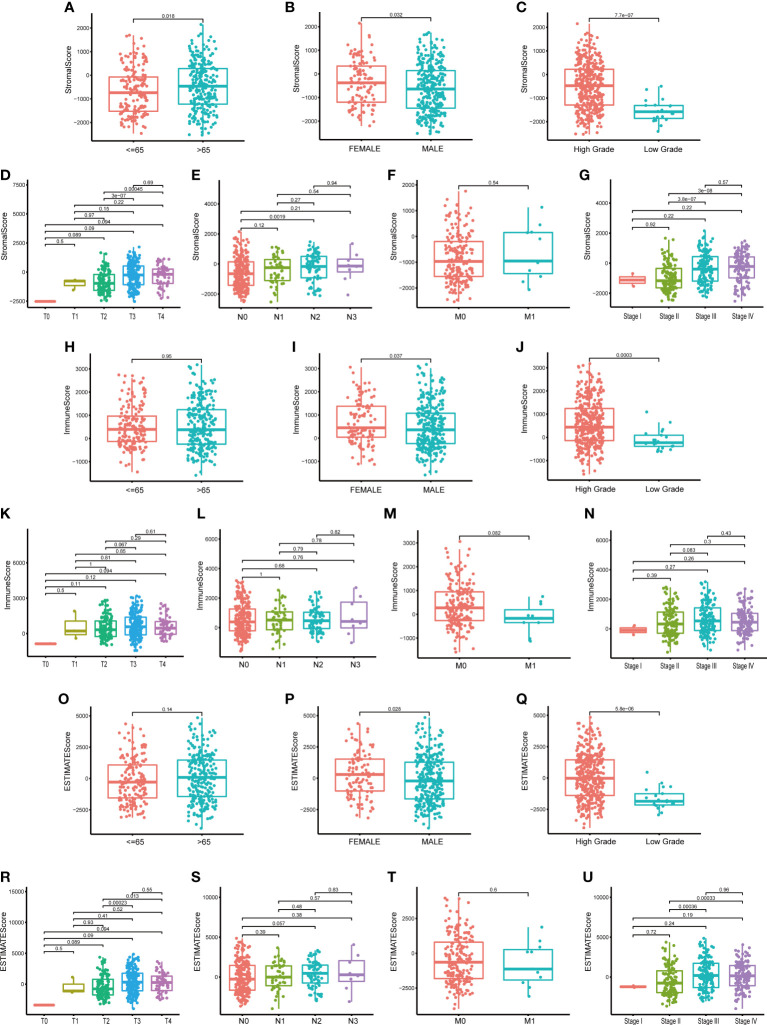
The relationship between Scores and clinic–pathological characteristics of BLCA patients. **(A–G)** The relationship between Stromal Score and clinic–pathological characteristics of BLCA patients, including age, gender, pathology grade, TNM classification and stage; **(H–N)** The relationship between Immune Score and clinic–pathological characteristics of BLCA patients; **(O–U)** The relationship between ESTIMATE Score and clinic–pathological characteristics of BLCA patients. P-value was showed in each plot.

To learn the association between the proportion of immune/stromal component in TME and the clinicopathological characteristics of BLCA patients, we set up the immune/stromal component evaluating system of TME in BLCA samples. Immune, Stromal and ESTIMATE Score represented the proportion of immune, stromal and the total component in TME of each tumor sample, respectively. The higher Score suggested the larger proportion of immune or stromal component in TME. The clinicopathological characteristics of BLCA patients, including age, gender, pathology grade, clinical stage and TNM classification, were concluded in **Supplement Table 1**. Difference analysis suggested that Stromal Score was associated with patients’ age and gender, especially the pathology grade (**Figures 2A–C**, p=0.018; 0.032; <0.001). The higher Stromal Score predicted the higher pathology grade of BLCA (**Figure 2C**, p<0.001). It was also found that Stromal Score was related with T, N classification and clinical stage of BLCA (**Figures 2D–G**). Regarding Immune Score, it was significantly up-regulated in female patients (**Figure 2I**, p=0.037) and the higher Immune Score suggested the higher pathology grade of BLCA (**Figures 2H–J**). However, neither TNM classification nor clinical stage of BLCA was associated with Immune Score (**Figures 2K–N**, p>0.05). As for ESTIMATE Score, the results showed that it was up-regulated in female patients and predicted the higher pathology grade of BLCA (**Figures 2O–Q**). In addition, ESTIMATE Score was connected with T classification and clinical stage of BLCA (**Figures 2R–U**). From above, we could conclude that the stromal and immune component in TME was significantly associated with the pathology grade of BLCA, and partly related with TNM classification and clinical stage. This provided a new sight for us to explore the underlying mechanisms of the development and progression of BLCA.

### DEGs were primarily enriched in immune-associated activities

**Figure 3:**
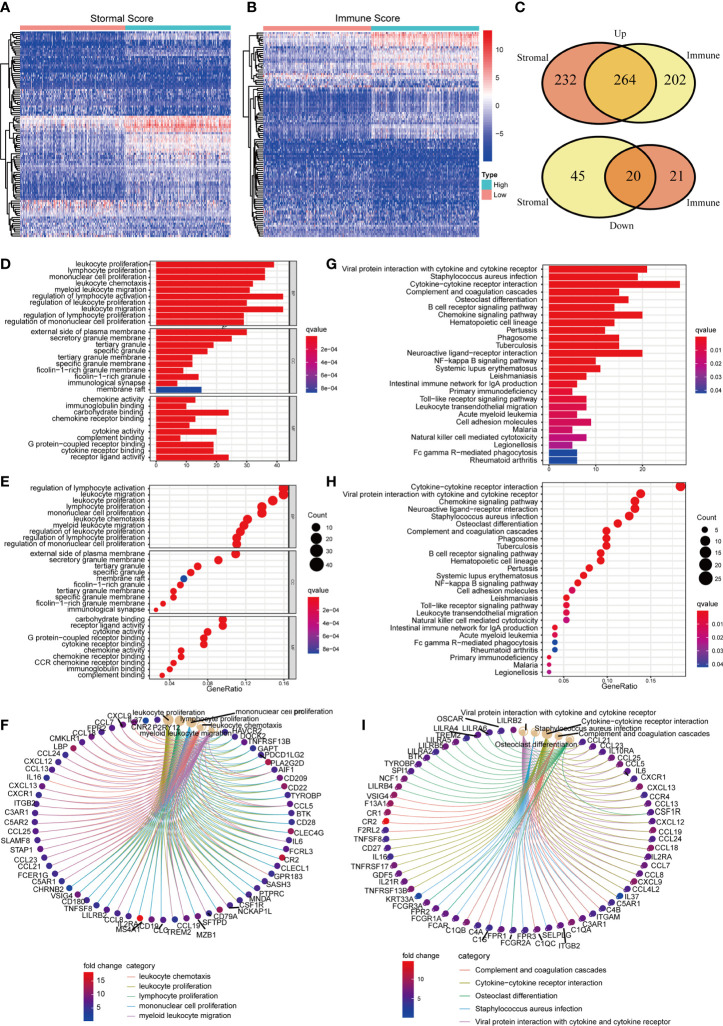
The identification and enrichment analysis of DEGs. **(A, B)** The heatmaps of DEGs obtained by comparison between high Stromal Score samples and low Stromal Score samples (or high Immune Score samples and low Immune Score). p<0.05, FDR<0.05 and log2^FC >^2 were set up to scan the DEGs; **(C)** Venn plots of DEGs which were commonly up-regulated or down-regulated in both Immune and Stromal Score; **(D–F)** GO enrichment analysis of 284 DEGs; p<0.05 was considered to be statistically significant; BP, biological process; CC, cell component; MF, molecular function; **(G–I)** KEGG enrichment analysis of 284 DEGs; p<0.05 was considered to be statistically significant.

In order to understand the underlying mechanisms of BLCA TME, we conducted difference analysis to acquire the DEGs profile in TME. BLCA samples were divided into two groups, including high Stromal/Immune Score group and low Stromal/Immune Score group. Heat map shows the DEGs profile in TME of BLCA (**Figures 3A, B**). To be specific, there were 537 DEGs obtained from the comparison between high Stromal Score and low Stromal Score, among which 496 DEGs were up-regulated and 41 DEGs were down-regulated (**Supplement Table 2**). Besides, there were 531 DEGs obtained from the comparison between high Immune Score and low Immune Score, among which 466 DEGs were up-regulated and 65 DEGs were down-regulated (**Supplement Table 3**). In conclusion, a total of 284 DEGs were synchronously up-regulated or down-regulated in Stromal or Immune group (**Figure 3C**; **Supplement Table 4**). Next, we carried out GO and KEGG enrichment analysis to assess the biological functions of these 284 DEGs. Go enrichment analysis revealed that 284 DEGs were primarily enriched in immune-associated activities, such as leukocyte proliferation, migration and chemotaxis, lymphocyte and mononuclear cell proliferation and so on (**Figures 3D–F**). Similarly, KEGG enrichment analysis suggested that these DEGs were mainly enriched in immune-associated activities, including complement and coagulation cascades, cytokine-cytokine receptor interaction, B cell receptor signaling pathway, chemokine signaling pathway and so on (**Figures 3G–I**). Therefore, we considered that DEGs acquired from Stromal and Immune group of BLCA were significantly associated with immune-associated activities in TME.

### PPI network and univariable COX regression analysis of 284 DEGs

**Figure 4:**
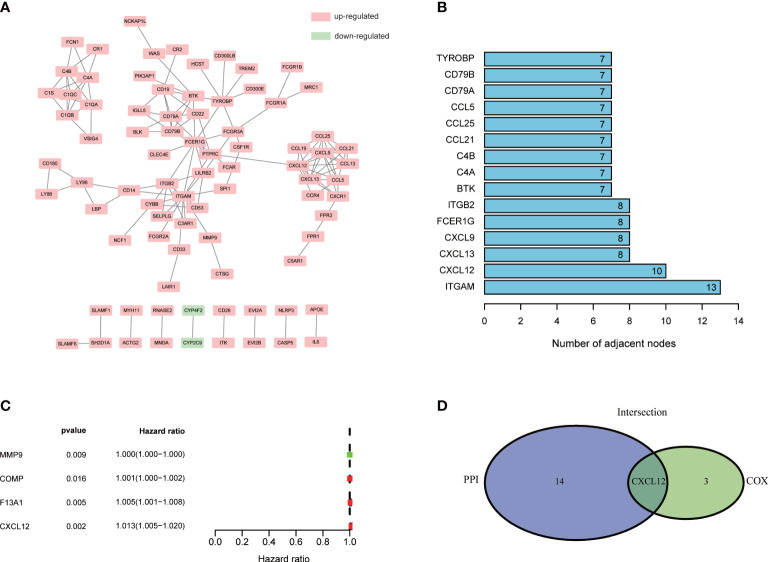
PPI network and univariable COX regression analysis of 284 DEGs. **(A)** PPI network was constructed by STRING database and Cytoscape software; interaction confidence value >0.95; **(B)** The top 10 genes which shared the leading nodes in PPI network; **(C)** Univariable COX regression analysis of 284 DEGs; p<0.05 was considered to be statistically significant; **(D)** Intersection analysis of DEGs which shared the leading nodes in PPI network and were closely related with BLCA patients’ survival. p<0.05 was considered to be statistically significant.

To learn the detailed reciprocity among these 284 DEGs, we plotted PPI network through STRING database and cytoscape software. The potential interactions among DEGs were shown in **Figure 4A**. DEGs, which shared more than seven nodes, were ranked in **Figure 4B** (**Supplement Table 5**). Univariable COX regression of 284 DEGs was conducted at the same time to find out the specific DEGs, which were significantly associated with BLCA patients’ survival (**Figure 4C**). MMP9, COMP, F13A1 and CXCL12 drew our attention (**Figure 4C**, p<0.05; **Supplement Table 6**). Intersection analysis was finally carried out to search the DEGs who shared the leading nodes in PPI network and were significantly related with BLCA patients’ survival (**Figure 4D**). Fortunately, CXCL12 emerged.

### CXCL12 was down-regulated in BLCA tissues and associated with the clinicopathological characteristics

**Figure 5:**
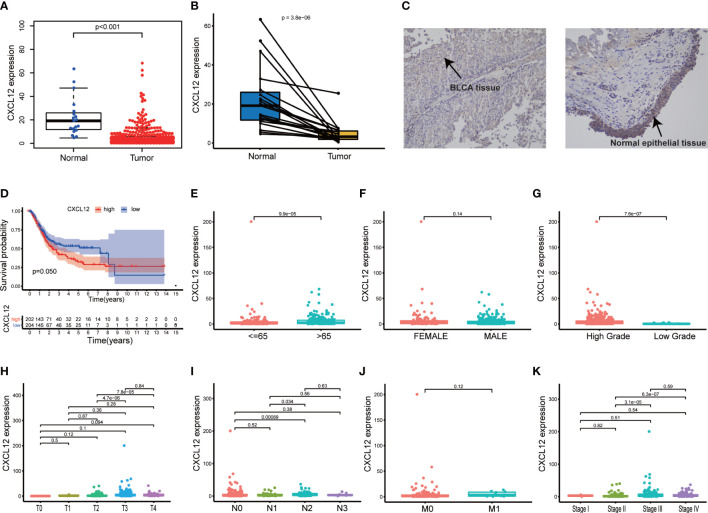
Single gene analysis of CXCL12 in BLCA. **(A)** Difference analysis of the expression of CXCL12 between BLCA samples and normal samples; **(B)** Paired analysis of the expression of CXCL12 between BLCA samples and paired normal samples; **(C)** The expression of CXCL12 in BLCA tissues and normal epithelial tissues; **(D)** Survival analysis of the expression of CXCL12 and the BLCA patients’ survival; patients were grouped into high CXCL12 group and low CXCL12 group, compared with the median; p value and the number of samples were displayed in the diagram; **(E–K)** The relationship between the expression of CXCL12 and clinic–pathological characteristics of BLCA patients, including age, gender, pathology grade, TNM classification and stage; p value was displayed in the diagram and p<0.05 was considered to be statistically significant.

The CXC chemokine CXCL12 participated greatly in multiple physiological and pathological processes through interacting with its receptors CXC chemokine receptor 4 (CXCR4) and atypical chemokine receptor 3 (ACKR3) (30). In our study, CXCL12 was significantly down-regulated in BLCA tissues compared with normal tissues (**Figure 5A**, p<0.001). Besides, the paired analysis found that CXCL12 was obviously down-regulated in BLCA tissues compared with the paired normal tissues (**Figure 5B**, p<0.001). In order to further validate the expression of CXCL12 in BLCA, we carried out IHC and the result was consistent with the above analysis (**Figure 5C**). However, survival analysis suggested that the expression of CXCL12 was not significantly associated with the survival of BLCA patients (**Figure 5D**, p=0.050). As for clinicopathological characteristics of BLCA patients, the results showed that the expression of CXCL12 varied with ages and higher expression of CXCL12 predicted the higher pathology grade of BLCA (**Figures 5E–G**; p<0.001, p<0.001). In addition, the expression of CXCL12 was partly related with T, N classification and clinical stages of BLCA (**Figures 5H–K**). Hence, we concluded that CXCL12 had the potential to participate in the progression of BLCA.

### CXCL12 participated greatly in immune-associated activities

**Figure 6:**
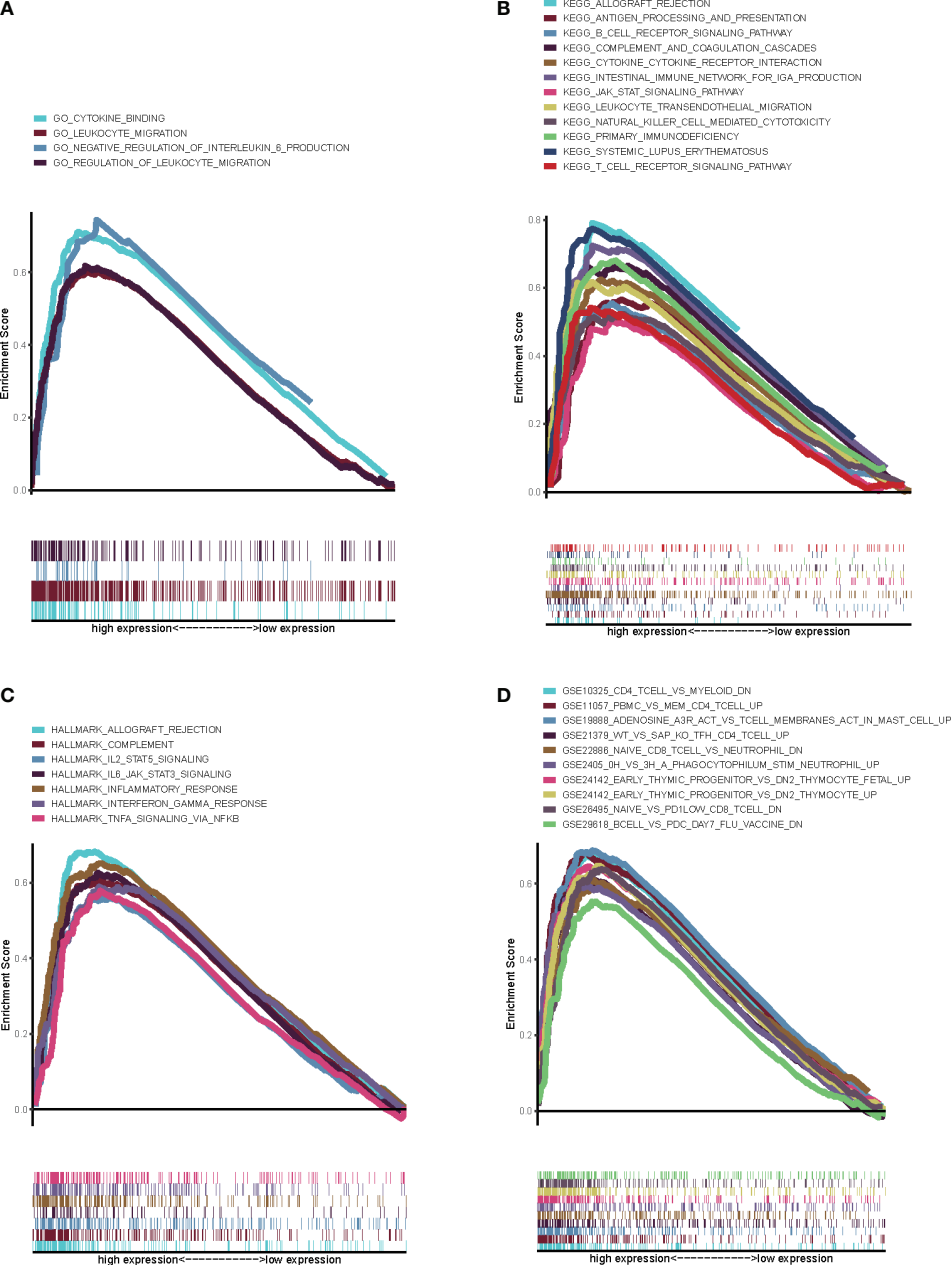
GSEA for genes in high CXCL12 expression group. **(A)** GSEA for genes in high CXCL12 expression group in C5 collection, the gene ontology sets; **(B)** GSEA for genes in high CXCL12 expression group in C2 collection, the KEGG gene sets database; **(C)** GSEA for genes in high CXCL12 expression group in hallmark gene sets; **(D)** GSEA for genes in high CXCL12 expression group in C7 collection defined by MSigDB, the immunologic gene sets. FDR<0.05 and p<0.05 were considered to be statistically significant.

On account of the potential role of CXCL12 in the progression of BLCA, we further explored the underlying mechanisms of CXCL12. BLCA samples were first divided into two groups, including high CXCL12 expression group and low CXCL12 expression group. GSEA suggested that for C5 collection, the gene ontology sets, genes in high CXCL12 expression group were enriched in cytokine binding, leukocyte migration and negative regulation of interleukin 6 production (**Figure 6A**, p<0.05). For C2 collection, the KEGG gene sets database, genes were enriched in allograft rejection, antigen processing and presentation, B cell receptor signaling pathway, complement and coagulation cascades, and so on (**Figure 6B**, p<0.05). For hallmark gene sets, genes in high CXCL12 expression group were primarily enriched in allograft rejection, complement, IL2 stats signaling, IL6 jak stat3 signaling and so on (**Figure 6C**, p<0.05). For C7 collection defined by MSigDB, the immunologic gene sets, genes were found to be enriched in multiple immune-associated activities, which were related with CD4 T cell, CD8 T cell, B cell and so on (**Figure 6D**, p<0.05). As a result, according to GSEA, CXCL12 was significantly related with immune-associated activities and had the potential to regulate TIME of BLCA.

### CXCL12 could communicate with TICs in TIME

**Figure 7:**
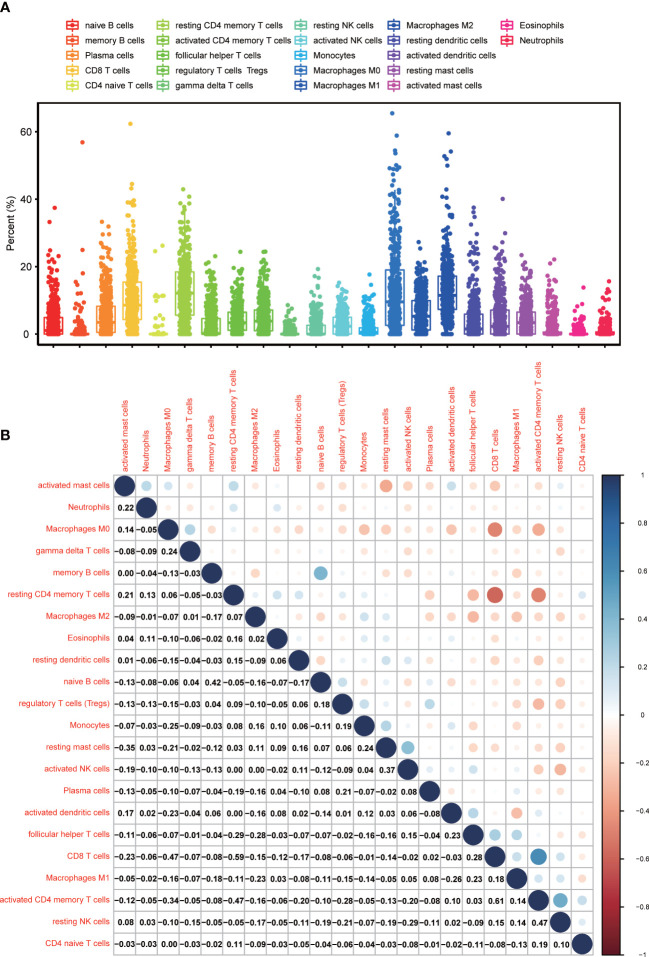
TICs Profile, and the difference analysis as well as correlation analysis between CXCL12 and TICs in TIME of BLCA. **(A)** The ratio of TICs in BLCA samples; **(B)** The correlation analysis among TICs; each spot indicated p value.

**Figure 8:**
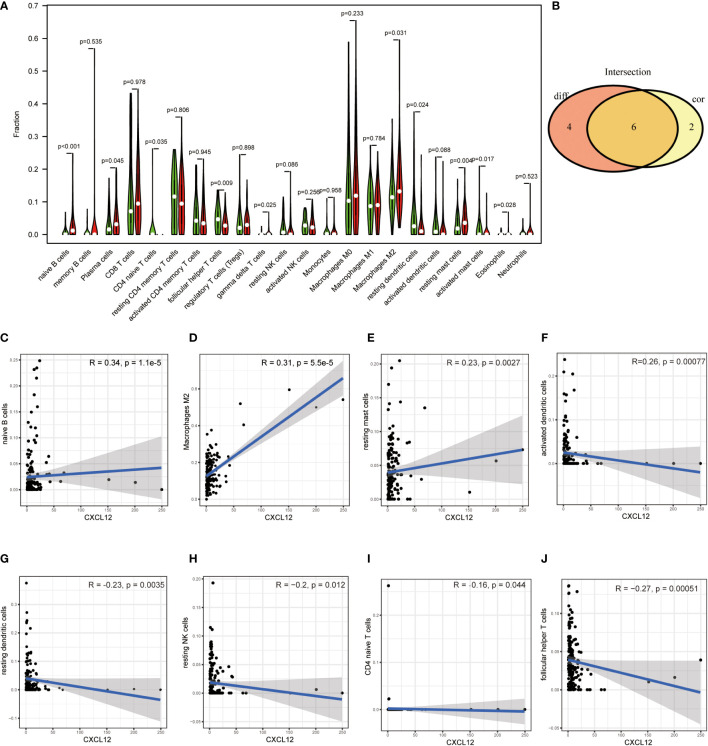
**(A)** Violin plot displayed the association between the expression of CXCL12 and TICs; p<0.05 was considered to be statistically significant; **(B)** Venn plot showed 6 TICs shared by the difference test **(A)** and correlation test **(C–J)**; **(C–J)** Scatter plots showed the association between the expression of CXCL12 and 8 TICs; p<0.05 was considered to be statistically significant.

Since GSEA suggested that CXCL12 was significantly correlated with immune-associated activities, we speculated that there could be underlying connections between CXCL12 and TIME of BLCA. In consequence, we calculated the proportion of TICs in BLCA samples through CIBERSORT algorithm. The specific ratio of 22 TICs in BLCA sample was shown in **Figure 7A**. And the association among these TICs was exhibited in **Figure 7B**. Difference analysis was carried out to learn the association between the expression of CXCL12 and specific TICs. The results showed that 10 kinds of TICs, such as Macrophages M2, naïve B cell, gamma delta T cells, CD4 naïve T cells, follicular helper T cells and so on, were significantly associated with the expression of CXCL12 (**Figure 8A**; **Supplement Table 7**). The correlation analysis suggested that the expression of CXCL12 were related with 8 kinds of TICs, among which naïve B cells, macrophages M2 and resting mast cells were positively associated, and activated dendritic cells, resting dendritic cells, resting NK cells, CD4 naïve T cells and follicular helper T cells were negatively associated (**Figures 8C–J**, p<0.05; **Supplement Table 7**). Finally, intersection analysis between difference analysis and correlation analysis suggested that 6 kinds of TICs were significantly related with the expression of CXCL12 (**Figure 8B**). In conclusion, CXCL12 was obviously related with various TICs in TIME of BLCA and had the potential to regulate the TIME of BLCA probably *via* communicating with multiple TICs.

## Discussion

In this study, we focused on the associations between IRGs and TICs in the TME of BLCA. Firstly, we downloaded the transcriptome profiling and clinical data of 414 BLCA samples and 19 normal samples from TCGA database, and calculated the proportion of immune and stromal component in TME of each BLCA samples with the help of ESTIMATE algorithm. Secondly, difference analysis found out that Immune/Stromal Score was associated with the clinicopathological characteristics of BLCA. Thirdly, DEGs were obtained through the comparison between high Immune Score and low Immune Score (or high Stromal Score and low Stromal Score), and GO and KEGG enrichment analysis suggested that these DEGs were mainly enriched in immune-related activities. Fourthly, PPI network and univariable COX regression analysis found that CXCL12 shared the leading nodes in PPI network and was significantly related with BLCA patients’ survival. Fifthly, signal gene analysis was conducted and found out that CXCL12 was down-regulated in BLCA samples compared with normal sample and significantly related with the clinicopathological characteristics of BLCA. GSEA revealed that CXCL12 was significantly associated with immune-associated activities and could play an important role in regulating TIME of BLCA. Finally, CIBERSORT algorithm was applied to calculate the proportion of TICs in BLCA samples, and difference analysis as well as correlation analysis suggested that CXCL12 was obviously connected with multiple TICs in TME of BLCA and could modulate the TIME of BLCA *via* closely communicating with TICs.

CXCL12 was traditionally classified as a homeostatic CXC chemokine and took a great part in modulating kinds of physiological and pathological processes *via* binding to its receptors CXCR4 and ACKR3 (30, 31). CXCL12/CXCR4 axis had been proved to be associated with the progression and therapy of cancers. For example, CXCL12/CXCR4 axis advanced the invasion and metastasis of pancreatic cancer through complex crosstalk with other pathways, and was correlated with the poor prognosis of patients (32). Treatment targeting the CXCL12/CXCR4 pathway increased the efficacy of radiotherapy of locally advanced cervical cancer (33). The role of CXCL12 in tumor development mainly depended on the specific microenvironment of tumors (34). In addition, CXCL12-CXCR4/CXCR7 axis had a great influence in gastrointestinal malignancies through immune resistance (35). Further researches suggested that the mechanisms of immunotherapy resistance could be associated with the CXCL12/CXCR4 axis (36). However, only a few studies explored the association between CXCL12 and tumorigenesis and progression of BLCA (37). Researches found that CXCL12 was down-regulated in BLCA tissues compared with normal bladder mucosal tissues, and positively associated with the differentiation degree and invasive depth of BLCA tissues (38). Additionally, CXCL12/CXCR4 promoted the invasion of BLCA cells through activating Stat3 (39).

Furthermore, increasing studies have revealed that TICs played an important role in the progression and treatment of BLCA. For example, intratumoral TIGIT+ CD8+ T-cell abundance functioned as a potential prognostic factor for patients’ survival and a predictive biomarker for adjuvant chemotherapeutic effect (40). CD19+ tumor-infiltrating B-cells activated CD4+ tumor-infiltrating T-cells in the TMB of BLCA and acted as an independent prognostic factor for post-surgery survival and adjuvant chemotherapy benefits of BLCA patients (41). Besides, DC-SIGN+ TAM infiltration was significantly associated with a tumor-promoting TIME and functioned as a prognostic indicator and therapeutic target in the immunotherapy of BLCA (42). However, there are few researches focusing on the associations between CXCL12 and TICs in TIME of BLCA.

In our study, we confirmed that CXCL12 was significantly down-regulated in BLCA tissues and associated with patients’ clinicopathological characteristics. In addition, functional analysis revealed that CXCL12 participated in immune-associated activities and could regulate TIME of BLCA probably through communicating with multiple TICs, such as macrophages M2, B cells naïve, T cells follicular helper, mast cells resting, dendritic cells resting and T cells CD4 naive. Further researches should be carried out to clarify the accuracy of the above combined analyses, and focused on the underlying mechanisms of the communication between CXCL12 and TICs in TIME of BLCA.

## Data availability statement

The original contributions presented in the study are included in the article/**Supplementary Materials**. Further inquiries can be directed to the corresponding author.

## Ethics statement

The studies involving human participants were reviewed and approved by The Ethics Committee of Nanjing Jiangning Hospital. The patients/participants provided their written informed consent to participate in this study.

## Author contributions

Conceptualization: JW; methodology: JW, SZ, DQ; software, validation, formal analysis, investigation, resources, data curation, writing—original draft preparation: JW, YX; Writing—review and editing, supervision and project administration: XH; Funding acquisition: JW and XH. All authors have read and agreed to the published version of the manuscript

## Funding

This research was funded by the National Nature Science Foundation of China, grant number 82103032, Medical Research Grant of Jiangsu Commission of Health, grant number M2020010, the Medical Science and Technology Development Foundation of Nanjing, grant number YKK21224, the Science Foundation of Jiangsu Health vocational college, grant number JKC201948, the Science and Technology Development Fund of Nanjing Medical University, grant number NMUB2019235, the Research and development fund of Kangda College of Nanjing Medical University, grant number KD2020KYJJZD006 and Youth Innovation Research Fund project of Jiangning Hospital, grant number JNYYZXKY202023.

## Conflict of interest

The authors declare that the research was conducted in the absence of any commercial or financial relationships that could be construed as a potential conflict of interest.

## Publisher’s note

All claims expressed in this article are solely those of the authors and do not necessarily represent those of their affiliated organizations, or those of the publisher, the editors and the reviewers. Any product that may be evaluated in this article, or claim that may be made by its manufacturer, is not guaranteed or endorsed by the publisher.
