# Supplementary material for: CXCL12, a potential modulator of tumor immune microenvironment (TIME) of bladder cancer: From a comprehensive analysis of TCGA database
Source: Front Oncol. 2022 Nov 7;12:1031706. doi: 10.3389/fonc.2022.1031706 (PMC9676933; doi:10.3389/fonc.2022.1031706)
Supplement: Supplementary file 1 [file Table_1.docx]

Supplement Table 1: The clinic–pathological characteristics of BC cases from TCGA database.

| Clinic–pathological characteristics | Classification | Total | Percentage (%) |
| --- | --- | --- | --- |
| Age | <=65 | 162 | 39.32% |
|  | >65 | 250 | 60.68% |
| Gender | Male | 304 | 73.79% |
|  | Female | 108 | 26.21% |
| Stage | I | 2 | 0.49% |
|  | II | 131 | 31.80% |
|  | III | 141 | 34.22% |
|  | IV | 136 | 33.01% |
|  | Unknow | 2 | 0.49% |
| T Classification | T0 | 1 | 0.24% |
|  | T1 | 3 | 0.73% |
|  | T2 | 120 | 29.13% |
|  | T3 | 196 | 47.57% |
|  | T4 | 59 | 14.32% |
|  | Unknow | 33 | 8.01% |
| N Classification | N0 | 239 | 58.01% |
|  | N1 | 47 | 11.41% |
|  | N2 | 76 | 18.45% |
|  | N3 | 8 | 1.94% |
|  | Unknow | 42 | 10.19% |
| M Classification | M0 | 196 | 47.57% |
|  | M1 | 11 | 2.67% |
|  | Unknow | 205 | 49.76% |
| Grade | High grade | 388 | 94.17% |
|  | low grade | 21 | 5.10% |
|  | Unknow | 3 | 0.73% |
