# Supplementary material for: CXCL12, a potential modulator of tumor immune microenvironment (TIME) of bladder cancer: From a comprehensive analysis of TCGA database
Source: Front Oncol. 2022 Nov 7;12:1031706. doi: 10.3389/fonc.2022.1031706 (PMC9676933; doi:10.3389/fonc.2022.1031706)
Supplement: Supplementary file 2 [file Table_2.docx]

Supplement Table 2: DEGs obtained from the comparison of high Stromal Scores and low Stromal Scores

| gene | conMean | treatMean | logFC | pValue | FDR |
| --- | --- | --- | --- | --- | --- |
| PDGFRB | 4.806406 | 21.7511 | 2.178058 | 3.36E-58 | 1.19E-54 |
| SCGB2A1 | 1.298567 | 0.198727 | -2.70806 | 5.32E-06 | 1.49E-05 |
| POPDC2 | 0.796002 | 3.356492 | 2.07611 | 7.83E-25 | 1.13E-23 |
| MARCO | 1.398389 | 8.758735 | 2.646957 | 1.98E-29 | 3.93E-28 |
| KCNMA1 | 0.189171 | 0.824483 | 2.123801 | 2.77E-37 | 9.68E-36 |
| C5AR1 | 1.628821 | 7.875877 | 2.273612 | 5.24E-51 | 1.56E-48 |
| SLAMF1 | 0.207295 | 1.1041 | 2.413114 | 3.13E-32 | 7.38E-31 |
| SIGLEC10 | 0.640789 | 3.606721 | 2.492766 | 2.08E-41 | 1.14E-39 |
| CHRNB2 | 0.269448 | 0.051791 | -2.37925 | 0.012111 | 0.018969 |
| SLCO2B1 | 0.995502 | 5.695268 | 2.516267 | 1.38E-48 | 2.18E-46 |
| MRVI1 | 1.132806 | 4.90991 | 2.115796 | 3.71E-41 | 1.99E-39 |
| HAVCR2 | 1.21406 | 5.210562 | 2.101599 | 5.55E-43 | 3.68E-41 |
| SCARF2 | 1.307588 | 6.011619 | 2.200846 | 8.56E-47 | 1.03E-44 |
| DOCK2 | 0.378076 | 1.951431 | 2.367784 | 8.07E-42 | 4.65E-40 |
| C2orf40 | 0.187655 | 1.385097 | 2.883831 | 7.97E-19 | 8.18E-18 |
| FCRL1 | 0.047771 | 0.392627 | 3.038944 | 3.23E-15 | 2.62E-14 |
| RASGRP4 | 0.132019 | 0.546474 | 2.049411 | 6.73E-45 | 6.01E-43 |
| CLSTN2 | 0.204312 | 1.098692 | 2.426942 | 2.86E-25 | 4.26E-24 |
| FCER1G | 10.29368 | 50.6063 | 2.297559 | 1.35E-44 | 1.14E-42 |
| TNFRSF13B | 0.061309 | 0.346227 | 2.497547 | 3.42E-21 | 3.99E-20 |
| SGCD | 0.115123 | 0.905696 | 2.975846 | 5.78E-52 | 2.04E-49 |
| CCL21 | 4.933589 | 24.24003 | 2.296682 | 1.90E-30 | 4.02E-29 |
| PI16 | 0.247989 | 4.353352 | 4.133779 | 2.69E-16 | 2.34E-15 |
| C20orf141 | 0.064496 | 0.472913 | 2.874284 | 1.42E-14 | 1.10E-13 |
| C4B | 0.286663 | 1.698771 | 2.567065 | 1.82E-40 | 8.97E-39 |
| FOLR2 | 2.54061 | 17.92041 | 2.818357 | 1.74E-44 | 1.45E-42 |
| ITGAM | 0.84298 | 3.524753 | 2.063952 | 2.12E-45 | 2.01E-43 |
| CHRM2 | 0.053186 | 0.487998 | 3.197762 | 1.31E-11 | 7.68E-11 |
| SPON2 | 3.459419 | 15.92853 | 2.203012 | 1.68E-46 | 1.93E-44 |
| DDR2 | 0.583152 | 3.607197 | 2.628935 | 1.12E-57 | 1.98E-54 |
| CCL23 | 0.179752 | 1.120181 | 2.639651 | 1.45E-32 | 3.51E-31 |
| IL21R | 0.281706 | 1.246971 | 2.146167 | 9.83E-35 | 2.73E-33 |
| P4HA3 | 0.419213 | 2.390671 | 2.51166 | 1.64E-49 | 3.05E-47 |
| FLNC | 1.315247 | 10.7164 | 3.026415 | 4.74E-37 | 1.62E-35 |
| HIST1H1B | 0.456246 | 7.050754 | 3.949895 | 0.00431 | 0.007361 |
| LMOD1 | 2.327696 | 20.8021 | 3.159755 | 3.21E-37 | 1.11E-35 |
| SIT1 | 0.766063 | 3.199563 | 2.062339 | 1.69E-26 | 2.74E-25 |
| COL8A1 | 1.528594 | 8.298216 | 2.440596 | 1.20E-45 | 1.16E-43 |
| PGM5 | 0.956368 | 4.388691 | 2.198154 | 1.38E-19 | 1.47E-18 |
| PLA2G5 | 0.171596 | 1.244312 | 2.858264 | 3.81E-44 | 2.92E-42 |
| F2RL2 | 0.569516 | 2.460568 | 2.111183 | 1.25E-26 | 2.04E-25 |
| FOXN4 | 0.660372 | 0.09688 | -2.76901 | 0.000205 | 0.000447 |
| SERTM2 | 0.019121 | 0.187132 | 3.290861 | 7.31E-22 | 8.92E-21 |
| ADAM33 | 0.380063 | 1.680432 | 2.144521 | 2.62E-26 | 4.18E-25 |
| LEFTY2 | 0.060069 | 0.516748 | 3.104758 | 1.15E-26 | 1.89E-25 |
| PRUNE2 | 0.327185 | 1.6661 | 2.348297 | 2.48E-35 | 7.30E-34 |
| COL10A1 | 0.979121 | 12.89528 | 3.719211 | 1.33E-44 | 1.14E-42 |
| GAPT | 0.117243 | 0.482421 | 2.040788 | 3.80E-28 | 6.96E-27 |
| GAS7 | 0.714895 | 3.098465 | 2.11575 | 1.10E-49 | 2.15E-47 |
| CTGF | 18.79372 | 109.1827 | 2.538422 | 1.26E-50 | 3.24E-48 |
| PLN | 0.723482 | 7.991188 | 3.465381 | 3.40E-42 | 2.08E-40 |
| P2RY12 | 0.053512 | 0.31126 | 2.540173 | 2.74E-20 | 3.02E-19 |
| STAB1 | 2.93246 | 13.82584 | 2.237184 | 1.26E-41 | 7.10E-40 |
| PYGM | 0.169643 | 0.682822 | 2.009008 | 5.52E-16 | 4.70E-15 |
| RXRG | 0.027257 | 0.127051 | 2.22069 | 6.60E-09 | 2.77E-08 |
| ASB5 | 0.056641 | 0.759677 | 3.745463 | 2.24E-26 | 3.59E-25 |
| CPB1 | 0.083047 | 0.508163 | 2.613293 | 0.000476 | 0.000975 |
| PDCD1LG2 | 0.770514 | 3.147128 | 2.030144 | 2.20E-37 | 7.73E-36 |
| STAP1 | 0.184857 | 1.208658 | 2.708922 | 1.12E-21 | 1.34E-20 |
| SCARA5 | 0.29687 | 1.389277 | 2.226432 | 3.39E-06 | 9.77E-06 |
| CLEC17A | 0.048002 | 0.323738 | 2.753659 | 4.67E-13 | 3.13E-12 |
| SSTR5 | 0.422327 | 0.081723 | -2.36954 | 8.11E-10 | 3.82E-09 |
| COL5A1 | 8.744497 | 53.55739 | 2.614638 | 2.57E-53 | 1.25E-50 |
| NEXN | 1.082077 | 4.658227 | 2.105979 | 2.03E-46 | 2.27E-44 |
| LILRB4 | 0.603644 | 4.148911 | 2.780962 | 6.59E-46 | 6.69E-44 |
| KRT33A | 4.333432 | 0.412841 | -3.39185 | 3.00E-05 | 7.46E-05 |
| SLC2A4 | 0.19013 | 1.025449 | 2.431201 | 1.36E-16 | 1.21E-15 |
| WDFY4 | 0.171151 | 0.999009 | 2.545226 | 2.20E-40 | 1.07E-38 |
| FMO2 | 0.121377 | 0.780925 | 2.685692 | 6.54E-36 | 2.02E-34 |
| VPREB3 | 0.611676 | 5.667765 | 3.211941 | 2.71E-10 | 1.36E-09 |
| SCRG1 | 0.061689 | 0.670722 | 3.442619 | 1.56E-22 | 1.97E-21 |
| VENTX | 0.117445 | 0.514711 | 2.131774 | 1.38E-40 | 6.90E-39 |
| DCN | 5.040804 | 37.43318 | 2.892592 | 5.04E-53 | 2.22E-50 |
| MMP2 | 30.43549 | 147.2018 | 2.273969 | 2.13E-46 | 2.36E-44 |
| SIGLEC7 | 0.197756 | 1.000239 | 2.338549 | 3.49E-46 | 3.65E-44 |
| MAP1A | 0.562932 | 2.376339 | 2.077709 | 2.89E-40 | 1.38E-38 |
| DACT3 | 0.410063 | 2.241594 | 2.450607 | 5.10E-37 | 1.74E-35 |
| CRISPLD2 | 3.015261 | 13.01688 | 2.110029 | 1.52E-47 | 2.13E-45 |
| RAMP1 | 4.137711 | 20.50553 | 2.309108 | 7.75E-33 | 1.91E-31 |
| FHL1 | 2.285738 | 12.20495 | 2.416735 | 1.44E-28 | 2.72E-27 |
| ADAMTS2 | 1.545713 | 10.40295 | 2.750649 | 4.47E-57 | 6.12E-54 |
| KCNMB1 | 0.422575 | 2.582163 | 2.611299 | 6.91E-41 | 3.59E-39 |
| BCL2A1 | 1.65624 | 6.898135 | 2.058295 | 2.93E-34 | 7.84E-33 |
| PTGDR | 0.044177 | 0.190747 | 2.110307 | 1.03E-41 | 5.84E-40 |
| CD248 | 6.347463 | 28.92773 | 2.188201 | 3.45E-49 | 6.01E-47 |
| COL1A2 | 48.14174 | 373.8912 | 2.957259 | 5.73E-59 | 4.04E-55 |
| SERPINA9 | 0.025788 | 0.309634 | 3.585771 | 7.57E-05 | 0.000177 |
| PLA2G2D | 0.296181 | 2.799014 | 3.240366 | 8.39E-27 | 1.39E-25 |
| WISP2 | 0.408858 | 2.664091 | 2.703971 | 4.21E-20 | 4.57E-19 |
| DCSTAMP | 0.03022 | 0.336475 | 3.476933 | 3.06E-34 | 8.15E-33 |
| NCR3 | 0.153831 | 0.619026 | 2.008653 | 1.10E-21 | 1.32E-20 |
| AIF1 | 5.331266 | 26.59324 | 2.31851 | 5.78E-45 | 5.27E-43 |
| TRH | 0.183156 | 2.057113 | 3.489473 | 0.000103 | 0.000236 |
| SLC13A2 | 0.2153 | 0.050544 | -2.09073 | 0.009847 | 0.015744 |
| HTR3A | 0.072534 | 0.966317 | 3.735777 | 9.37E-08 | 3.38E-07 |
| THRSP | 0.071055 | 1.758496 | 4.629255 | 0.000205 | 0.000447 |
| FCN1 | 0.204909 | 1.186416 | 2.533555 | 3.93E-35 | 1.14E-33 |
| AEBP1 | 14.01682 | 113.5004 | 3.017466 | 2.03E-57 | 3.18E-54 |
| NCF1 | 0.251522 | 1.531145 | 2.605854 | 5.61E-37 | 1.90E-35 |
| CSDC2 | 0.341448 | 2.268002 | 2.731684 | 1.39E-38 | 5.48E-37 |
| KCNE4 | 0.515694 | 2.748868 | 2.414251 | 3.58E-53 | 1.63E-50 |
| SPI1 | 3.562815 | 16.67305 | 2.226429 | 8.89E-45 | 7.79E-43 |
| TPM2 | 23.84911 | 99.08668 | 2.054756 | 1.86E-38 | 7.28E-37 |
| CD209 | 0.564824 | 4.436613 | 2.973585 | 6.02E-39 | 2.48E-37 |
| IL10RA | 0.992142 | 4.620852 | 2.21954 | 7.25E-43 | 4.71E-41 |
| MEDAG | 0.934748 | 6.60106 | 2.820049 | 3.36E-42 | 2.06E-40 |
| GYPC | 1.289028 | 7.604997 | 2.560664 | 1.93E-58 | 9.06E-55 |
| POU4F3 | 1.173777 | 0.03537 | -5.05251 | 0.004383 | 0.007481 |
| MYH11 | 4.947159 | 56.55392 | 3.514955 | 5.29E-24 | 7.28E-23 |
| NFAM1 | 0.455909 | 2.111603 | 2.21152 | 8.44E-42 | 4.84E-40 |
| HSD11B1 | 0.613985 | 3.231459 | 2.395911 | 1.97E-41 | 1.08E-39 |
| GPR34 | 0.65087 | 3.044173 | 2.22561 | 8.71E-41 | 4.49E-39 |
| PDLIM3 | 1.059145 | 7.773872 | 2.875733 | 6.64E-50 | 1.42E-47 |
| CD70 | 0.530243 | 2.435155 | 2.199288 | 2.26E-24 | 3.18E-23 |
| SSC5D | 0.50326 | 3.463688 | 2.782935 | 8.58E-50 | 1.73E-47 |
| IHH | 3.344435 | 0.577992 | -2.53264 | 0.022734 | 0.033672 |
| CD22 | 0.206532 | 2.142576 | 3.374912 | 5.69E-29 | 1.10E-27 |
| SLA | 0.723026 | 3.250263 | 2.168436 | 2.92E-44 | 2.32E-42 |
| DPT | 0.691491 | 11.88414 | 4.103182 | 1.79E-46 | 2.02E-44 |
| AARD | 0.095392 | 0.536361 | 2.491269 | 1.36E-23 | 1.81E-22 |
| TYROBP | 14.16793 | 72.28133 | 2.350994 | 1.03E-47 | 1.46E-45 |
| CD300C | 0.316047 | 1.674841 | 2.405811 | 7.13E-42 | 4.18E-40 |
| GLYATL2 | 0.097205 | 0.783013 | 3.009928 | 5.10E-10 | 2.47E-09 |
| EVI2B | 1.4669 | 6.648599 | 2.18028 | 3.59E-43 | 2.47E-41 |
| PCOLCE | 7.399593 | 34.78361 | 2.23289 | 1.98E-47 | 2.64E-45 |
| BNC2 | 0.132446 | 0.799843 | 2.594318 | 1.20E-52 | 4.99E-50 |
| SYNM | 1.483004 | 12.45681 | 3.07034 | 5.21E-24 | 7.18E-23 |
| POU3F2 | 0.518734 | 0.032622 | -3.99107 | 0.017372 | 0.026323 |
| GPR84 | 0.364384 | 1.49278 | 2.034469 | 7.64E-29 | 1.46E-27 |
| SLAMF8 | 1.582872 | 8.236029 | 2.379404 | 1.07E-44 | 9.32E-43 |
| CCL25 | 0.034272 | 0.208488 | 2.604873 | 1.29E-08 | 5.21E-08 |
| THBS2 | 11.08026 | 50.5309 | 2.189174 | 3.52E-40 | 1.68E-38 |
| GDF5 | 0.030787 | 0.252199 | 3.034156 | 2.19E-15 | 1.80E-14 |
| CCL5 | 14.08558 | 82.4739 | 2.549719 | 2.03E-26 | 3.28E-25 |
| TMEM119 | 1.850605 | 9.767004 | 2.399919 | 1.13E-44 | 9.81E-43 |
| LUM | 33.37633 | 188.941 | 2.501039 | 1.13E-50 | 2.95E-48 |
| RANBP3L | 0.09622 | 0.406443 | 2.078651 | 1.85E-11 | 1.06E-10 |
| MRO | 0.039155 | 0.223309 | 2.511781 | 2.38E-38 | 9.18E-37 |
| CXorf21 | 0.24884 | 1.08785 | 2.128191 | 7.97E-41 | 4.12E-39 |
| TNFAIP8L2 | 1.201817 | 4.926524 | 2.035353 | 6.02E-39 | 2.48E-37 |
| C5AR2 | 0.105833 | 0.724035 | 2.774275 | 8.40E-43 | 5.41E-41 |
| BTK | 0.381478 | 1.962164 | 2.362773 | 7.82E-45 | 6.90E-43 |
| CD28 | 0.193302 | 0.819024 | 2.083047 | 4.67E-38 | 1.76E-36 |
| C3AR1 | 1.383848 | 7.842093 | 2.502553 | 3.09E-48 | 4.68E-46 |
| SIGLEC1 | 0.662122 | 3.812467 | 2.525556 | 2.23E-39 | 9.67E-38 |
| FMO1 | 0.186428 | 1.160624 | 2.638207 | 7.55E-42 | 4.38E-40 |
| NTS | 12.55681 | 1.55297 | -3.01537 | 0.002099 | 0.003805 |
| HSPB2 | 0.193746 | 0.86382 | 2.156567 | 1.28E-41 | 7.12E-40 |
| CLEC4G | 0.136652 | 1.578934 | 3.530376 | 8.06E-21 | 9.18E-20 |
| C1QA | 32.14243 | 206.7053 | 2.685025 | 6.92E-44 | 5.09E-42 |
| CRYAB | 2.802089 | 11.53452 | 2.041383 | 4.86E-35 | 1.40E-33 |
| KCNK12 | 0.055276 | 0.2385 | 2.109254 | 1.85E-07 | 6.39E-07 |
| MMP9 | 7.29999 | 94.61527 | 3.696107 | 4.19E-32 | 9.83E-31 |
| GDF10 | 0.034224 | 0.143384 | 2.066808 | 9.14E-27 | 1.51E-25 |
| EVI2A | 0.840642 | 4.289808 | 2.35135 | 4.33E-52 | 1.61E-49 |
| AC136428.1 | 0.089838 | 0.738371 | 3.038948 | 9.35E-21 | 1.06E-19 |
| TPSB2 | 2.109686 | 9.510942 | 2.17256 | 5.93E-27 | 9.94E-26 |
| FBN1 | 1.406075 | 7.644589 | 2.442766 | 5.36E-53 | 2.29E-50 |
| CDH11 | 0.884038 | 5.598151 | 2.66277 | 3.93E-55 | 3.08E-52 |
| ST6GALNAC5 | 0.377503 | 1.627186 | 2.10782 | 5.78E-43 | 3.81E-41 |
| FCRL2 | 0.039704 | 0.333572 | 3.070641 | 1.07E-21 | 1.29E-20 |
| ACTL6B | 0.474107 | 0.015154 | -4.96742 | 0.008376 | 0.013554 |
| TCL1A | 0.146589 | 2.570549 | 4.132229 | 2.23E-16 | 1.96E-15 |
| ITGB2 | 3.135646 | 16.67787 | 2.4111 | 3.01E-44 | 2.37E-42 |
| TACR2 | 0.343753 | 1.395919 | 2.021772 | 7.93E-05 | 0.000185 |
| PRKG1 | 0.318714 | 1.343555 | 2.07572 | 4.03E-45 | 3.75E-43 |
| CCL11 | 1.779426 | 10.21998 | 2.521909 | 8.17E-39 | 3.31E-37 |
| LY86 | 1.235139 | 5.444826 | 2.140212 | 1.70E-47 | 2.30E-45 |
| LAX1 | 0.152539 | 0.67962 | 2.155548 | 1.59E-31 | 3.56E-30 |
| FDCSP | 3.078565 | 108.8216 | 5.143563 | 1.92E-15 | 1.58E-14 |
| P2RX1 | 0.274849 | 2.752108 | 3.323824 | 4.17E-39 | 1.76E-37 |
| SELPLG | 2.492502 | 12.03105 | 2.271096 | 1.68E-46 | 1.93E-44 |
| CD84 | 0.28768 | 1.54228 | 2.422527 | 6.50E-45 | 5.84E-43 |
| IL6 | 1.262978 | 9.11854 | 2.851973 | 7.02E-32 | 1.61E-30 |
| PTGER3 | 0.162541 | 0.739587 | 2.185918 | 5.49E-37 | 1.86E-35 |
| CYP4F8 | 16.52194 | 3.940999 | -2.06775 | 9.36E-08 | 3.38E-07 |
| SMOC2 | 2.400469 | 12.13838 | 2.338187 | 3.85E-35 | 1.12E-33 |
| ITGA11 | 0.608018 | 5.406832 | 3.152598 | 3.31E-54 | 1.95E-51 |
| GLT8D2 | 0.977306 | 5.0487 | 2.369029 | 2.42E-56 | 2.44E-53 |
| BHMT2 | 0.131672 | 0.637324 | 2.275075 | 2.02E-28 | 3.78E-27 |
| OMD | 0.090858 | 1.057552 | 3.540965 | 1.09E-46 | 1.29E-44 |
| ADAMTS16 | 0.232947 | 1.017693 | 2.127227 | 6.59E-42 | 3.88E-40 |
| CHRDL1 | 0.406213 | 2.76844 | 2.768764 | 7.38E-20 | 7.96E-19 |
| C1QC | 28.22583 | 162.9565 | 2.529399 | 2.58E-43 | 1.82E-41 |
| COL6A2 | 40.61092 | 211.6406 | 2.381677 | 3.23E-54 | 1.95E-51 |
| FCRL3 | 0.080785 | 0.459336 | 2.507391 | 7.84E-26 | 1.20E-24 |
| TPSD1 | 0.214691 | 1.192871 | 2.474104 | 3.63E-13 | 2.45E-12 |
| SH2D1A | 0.316576 | 1.311024 | 2.050072 | 1.16E-28 | 2.19E-27 |
| FNDC1 | 0.343956 | 5.961234 | 4.115317 | 3.15E-54 | 1.95E-51 |
| COMP | 2.997872 | 47.65206 | 3.990528 | 4.42E-38 | 1.67E-36 |
| SPARC | 87.37505 | 400.1566 | 2.195271 | 5.00E-58 | 1.41E-54 |
| CCDC187 | 0.294136 | 0.069321 | -2.08513 | 2.89E-08 | 1.12E-07 |
| ACKR1 | 2.706282 | 13.03606 | 2.268124 | 1.76E-13 | 1.23E-12 |
| SCIMP | 0.214247 | 1.11104 | 2.374565 | 4.22E-39 | 1.78E-37 |
| TAGLN | 22.17957 | 167.4788 | 2.916675 | 4.11E-50 | 8.93E-48 |
| COL8A2 | 1.602689 | 6.884926 | 2.102946 | 4.47E-44 | 3.41E-42 |
| COL5A2 | 9.828979 | 54.15894 | 2.462086 | 5.36E-52 | 1.94E-49 |
| AOX1 | 0.171587 | 1.042362 | 2.602841 | 3.01E-33 | 7.64E-32 |
| GEM | 2.191765 | 10.56505 | 2.269134 | 2.57E-41 | 1.40E-39 |
| CD48 | 1.045644 | 5.247615 | 2.32727 | 1.54E-34 | 4.21E-33 |
| XPNPEP2 | 0.153166 | 0.627206 | 2.033846 | 6.04E-32 | 1.39E-30 |
| CR2 | 0.286941 | 4.089617 | 3.833142 | 6.79E-11 | 3.67E-10 |
| CLEC4E | 0.203557 | 1.362536 | 2.742787 | 3.11E-41 | 1.67E-39 |
| PIK3AP1 | 0.880069 | 3.670501 | 2.060288 | 9.93E-38 | 3.60E-36 |
| BOC | 0.348262 | 1.917814 | 2.461219 | 6.44E-47 | 7.97E-45 |
| PRRX1 | 1.086852 | 6.490087 | 2.578083 | 2.27E-53 | 1.14E-50 |
| CLECL1 | 0.109851 | 0.615123 | 2.485321 | 9.37E-21 | 1.06E-19 |
| CXCR1 | 0.089162 | 0.459144 | 2.364453 | 1.89E-10 | 9.69E-10 |
| CIDEC | 0.088167 | 0.923063 | 3.38812 | 4.61E-11 | 2.53E-10 |
| FPR3 | 1.908326 | 9.848596 | 2.36761 | 1.01E-43 | 7.27E-42 |
| GPR183 | 1.967507 | 9.103654 | 2.210077 | 4.12E-46 | 4.24E-44 |
| ACTG2 | 11.09354 | 113.255 | 3.351784 | 3.40E-35 | 9.94E-34 |
| TNFAIP6 | 1.006621 | 6.189759 | 2.620363 | 1.83E-50 | 4.44E-48 |
| LRRC15 | 0.336062 | 6.002014 | 4.158647 | 1.76E-47 | 2.36E-45 |
| ACTC1 | 1.114526 | 13.24633 | 3.571091 | 6.23E-31 | 1.36E-29 |
| KRT81 | 3.417401 | 40.51018 | 3.567313 | 7.06E-08 | 2.59E-07 |
| MRC1 | 1.00471 | 7.982202 | 2.990007 | 2.48E-46 | 2.71E-44 |
| CASP5 | 0.091747 | 0.397239 | 2.114272 | 5.17E-17 | 4.75E-16 |
| HMHB1 | 0.062693 | 0.254884 | 2.023473 | 4.98E-12 | 3.04E-11 |
| LAIR1 | 0.783828 | 3.831686 | 2.289371 | 5.39E-47 | 6.73E-45 |
| PPY | 0.086394 | 1.184699 | 3.777448 | 0.000331 | 0.000696 |
| FCAMR | 0.043675 | 0.576369 | 3.722116 | 1.79E-05 | 4.62E-05 |
| CXCL13 | 5.298399 | 34.9218 | 2.720499 | 7.88E-22 | 9.58E-21 |
| SFRP4 | 1.809262 | 37.40161 | 4.369627 | 3.34E-50 | 7.59E-48 |
| ADAM12 | 0.973837 | 5.953645 | 2.61202 | 1.44E-51 | 4.83E-49 |
| TCAP | 2.862214 | 0.684084 | -2.06489 | 1.39E-06 | 4.23E-06 |
| MXRA8 | 8.20127 | 36.15363 | 2.140221 | 2.65E-45 | 2.48E-43 |
| BIRC7 | 0.134933 | 0.682559 | 2.338713 | 8.73E-24 | 1.18E-22 |
| LY9 | 0.07551 | 0.379094 | 2.327824 | 2.24E-32 | 5.33E-31 |
| ADAMTS4 | 1.143359 | 5.107548 | 2.159353 | 3.18E-46 | 3.37E-44 |
| REG4 | 1.267361 | 0.261465 | -2.27714 | 5.67E-08 | 2.11E-07 |
| COL6A1 | 34.70911 | 163.0584 | 2.232002 | 4.11E-50 | 8.93E-48 |
| FCGR2A | 2.26649 | 10.75924 | 2.247044 | 6.75E-46 | 6.80E-44 |
| COL11A1 | 0.594678 | 9.052509 | 3.928137 | 4.04E-41 | 2.14E-39 |
| KRT86 | 1.299849 | 6.593458 | 2.342691 | 0.000468 | 0.000958 |
| OLFML2B | 3.554987 | 16.64211 | 2.226922 | 9.03E-51 | 2.45E-48 |
| GXYLT2 | 0.475178 | 2.234544 | 2.233439 | 7.51E-47 | 9.22E-45 |
| SULF1 | 2.434838 | 21.19721 | 3.121976 | 2.41E-54 | 1.62E-51 |
| TGFB3 | 1.607306 | 7.747828 | 2.269147 | 1.94E-51 | 6.22E-49 |
| CR1 | 0.045579 | 0.449109 | 3.300623 | 5.35E-38 | 1.99E-36 |
| CD37 | 1.511248 | 7.512439 | 2.31354 | 1.33E-40 | 6.66E-39 |
| SASH3 | 1.749872 | 8.061912 | 2.203873 | 1.15E-37 | 4.14E-36 |
| MSRB3 | 1.429506 | 7.384848 | 2.369051 | 7.14E-50 | 1.48E-47 |
| LILRA2 | 0.065334 | 0.296026 | 2.179808 | 9.22E-44 | 6.67E-42 |
| FGF5 | 0.057255 | 0.27714 | 2.275136 | 1.30E-24 | 1.86E-23 |
| ABCC9 | 0.176046 | 0.708281 | 2.008373 | 1.50E-40 | 7.45E-39 |
| CCR4 | 0.225182 | 0.925714 | 2.039471 | 9.46E-31 | 2.03E-29 |
| GZMH | 1.527645 | 6.502742 | 2.089739 | 6.25E-22 | 7.66E-21 |
| TNFRSF17 | 0.250999 | 1.545954 | 2.622746 | 1.05E-25 | 1.61E-24 |
| PTPRC | 1.222571 | 5.909573 | 2.273135 | 1.82E-35 | 5.42E-34 |
| TPSAB1 | 2.118265 | 9.154114 | 2.111537 | 1.06E-32 | 2.59E-31 |
| KIAA1755 | 0.164558 | 0.970974 | 2.560833 | 1.12E-49 | 2.17E-47 |
| ADAMTS14 | 0.81677 | 3.482067 | 2.091943 | 1.24E-40 | 6.30E-39 |
| GREM1 | 0.915551 | 7.920384 | 3.112857 | 2.95E-50 | 6.83E-48 |
| ADAMDEC1 | 0.82609 | 4.601776 | 2.47782 | 3.96E-31 | 8.75E-30 |
| NUGGC | 0.086788 | 0.420105 | 2.275182 | 9.93E-19 | 1.01E-17 |
| SEMG1 | 1.254491 | 0.156667 | -3.00133 | 0.011058 | 0.017455 |
| MNDA | 1.00229 | 5.008454 | 2.321065 | 1.04E-40 | 5.30E-39 |
| CASQ2 | 0.337912 | 3.91459 | 3.534142 | 4.64E-24 | 6.42E-23 |
| CLEC4D | 0.032204 | 0.199592 | 2.63175 | 5.52E-31 | 1.21E-29 |
| FABP3 | 12.04817 | 50.54118 | 2.068645 | 0.000648 | 0.001289 |
| BLK | 0.104271 | 0.932912 | 3.161407 | 2.89E-19 | 3.05E-18 |
| ABI3BP | 0.370027 | 1.752513 | 2.243724 | 7.32E-30 | 1.49E-28 |
| FAM129C | 0.067675 | 0.592073 | 3.12909 | 1.34E-10 | 7.01E-10 |
| COL3A1 | 71.1757 | 620.639 | 3.124298 | 4.77E-57 | 6.12E-54 |
| HK3 | 0.653634 | 3.503725 | 2.422335 | 4.75E-39 | 1.99E-37 |
| HAND1 | 0.093394 | 0.488818 | 2.387898 | 9.11E-17 | 8.21E-16 |
| LILRB5 | 0.2107 | 1.305521 | 2.63136 | 6.63E-38 | 2.45E-36 |
| FMOD | 3.844918 | 19.48154 | 2.341083 | 1.13E-50 | 2.95E-48 |
| MFAP4 | 9.429984 | 49.65835 | 2.396709 | 6.55E-33 | 1.63E-31 |
| HAND2 | 0.30436 | 1.539668 | 2.338766 | 1.95E-32 | 4.68E-31 |
| TNXB | 0.253744 | 1.168955 | 2.203771 | 4.73E-23 | 6.11E-22 |
| LMCD1 | 1.408299 | 6.121635 | 2.119963 | 1.15E-46 | 1.35E-44 |
| CCER2 | 9.793015 | 0.342386 | -4.83806 | 4.51E-05 | 0.000109 |
| SLURP1 | 8.022709 | 0.903273 | -3.15086 | 0.000612 | 0.001224 |
| CNN1 | 5.496502 | 63.77699 | 3.53645 | 1.62E-39 | 7.19E-38 |
| CD300LF | 0.448479 | 1.816522 | 2.018067 | 8.07E-42 | 4.65E-40 |
| NTM | 0.562956 | 2.511779 | 2.157614 | 2.30E-42 | 1.44E-40 |
| AOAH | 0.540054 | 3.214727 | 2.573521 | 4.74E-40 | 2.22E-38 |
| FGL2 | 1.665407 | 8.084829 | 2.279342 | 3.68E-40 | 1.74E-38 |
| SLAMF6 | 0.440952 | 2.094342 | 2.247802 | 2.55E-34 | 6.85E-33 |
| IL16 | 0.336549 | 1.396965 | 2.053407 | 6.91E-46 | 6.91E-44 |
| MYLK | 1.545305 | 8.572416 | 2.47181 | 2.79E-44 | 2.24E-42 |
| WAS | 1.85123 | 7.602258 | 2.037943 | 4.90E-40 | 2.29E-38 |
| RHOH | 0.258293 | 1.306326 | 2.338434 | 1.00E-35 | 3.05E-34 |
| CCL13 | 1.828362 | 10.97494 | 2.58559 | 1.44E-26 | 2.35E-25 |
| ELN | 2.519472 | 11.13781 | 2.144272 | 1.62E-21 | 1.94E-20 |
| P2RY13 | 0.287665 | 1.288095 | 2.162776 | 1.32E-30 | 2.82E-29 |
| COL6A3 | 7.759386 | 49.562 | 2.67522 | 2.86E-59 | 4.03E-55 |
| TFF1 | 52.51155 | 9.055726 | -2.53573 | 8.52E-07 | 2.66E-06 |
| IBSP | 0.561084 | 3.185806 | 2.505369 | 3.07E-08 | 1.18E-07 |
| CPEB1 | 0.041593 | 0.188326 | 2.178807 | 2.14E-36 | 6.87E-35 |
| LILRA5 | 0.462598 | 2.717627 | 2.554516 | 2.27E-40 | 1.10E-38 |
| MAP1LC3C | 0.039181 | 0.270952 | 2.789812 | 1.04E-24 | 1.50E-23 |
| EMILIN1 | 5.46232 | 40.5295 | 2.891387 | 8.84E-58 | 1.98E-54 |
| FIBIN | 0.770246 | 6.443977 | 3.06456 | 3.87E-50 | 8.67E-48 |
| FPR1 | 0.65734 | 4.748665 | 2.85281 | 2.98E-49 | 5.33E-47 |
| CSMD2 | 0.044702 | 0.182942 | 2.032981 | 1.69E-40 | 8.33E-39 |
| DPEP3 | 0.059281 | 0.738603 | 3.639163 | 4.05E-10 | 2.00E-09 |
| MEFV | 0.122989 | 0.531006 | 2.110197 | 5.52E-23 | 7.12E-22 |
| LCN6 | 0.029786 | 0.209011 | 2.81088 | 1.63E-15 | 1.35E-14 |
| NPHS2 | 0.509808 | 0.094715 | -2.4283 | 0.000249 | 0.000534 |
| PTGFR | 0.157093 | 0.822653 | 2.388662 | 1.45E-34 | 3.99E-33 |
| CSF1R | 2.647699 | 15.03091 | 2.505122 | 2.37E-44 | 1.93E-42 |
| ITGBL1 | 0.37587 | 1.993619 | 2.407085 | 5.73E-36 | 1.77E-34 |
| OLFML1 | 0.519752 | 2.331572 | 2.165409 | 7.90E-56 | 7.43E-53 |
| SFRP1 | 1.218296 | 5.262336 | 2.110838 | 2.26E-26 | 3.62E-25 |
| CYBB | 2.258407 | 15.17474 | 2.748294 | 4.96E-44 | 3.72E-42 |
| LRRC25 | 0.759478 | 3.7545 | 2.30554 | 9.04E-46 | 8.87E-44 |
| FCER2 | 0.101803 | 1.422483 | 3.804561 | 9.01E-16 | 7.55E-15 |
| HAS1 | 0.139839 | 2.02362 | 3.855097 | 9.22E-31 | 1.98E-29 |
| SGCA | 0.380508 | 2.57476 | 2.758439 | 5.66E-24 | 7.78E-23 |
| CTSE | 36.37546 | 8.597564 | -2.08097 | 0.000485 | 0.000992 |
| THY1 | 7.258481 | 31.819 | 2.132149 | 9.32E-54 | 4.87E-51 |
| F13A1 | 1.741372 | 17.10149 | 3.295826 | 4.85E-39 | 2.03E-37 |
| SHISAL1 | 0.135132 | 0.919929 | 2.767158 | 8.99E-43 | 5.77E-41 |
| COL14A1 | 1.500701 | 7.824273 | 2.38232 | 4.15E-38 | 1.58E-36 |
| NCKAP1L | 0.704664 | 3.653917 | 2.374437 | 6.96E-45 | 6.18E-43 |
| HEPACAM2 | 2.008117 | 0.186086 | -3.4318 | 0.005394 | 0.00905 |
| MPEG1 | 1.597919 | 8.498634 | 2.411037 | 4.69E-45 | 4.33E-43 |
| FAM20A | 0.716318 | 3.32394 | 2.214222 | 1.39E-46 | 1.62E-44 |
| CD79A | 1.683293 | 16.82916 | 3.321604 | 1.79E-27 | 3.07E-26 |
| SFTPD | 0.517187 | 2.696277 | 2.382211 | 0.000467 | 0.000958 |
| GABRP | 2.624791 | 11.56328 | 2.139276 | 6.35E-06 | 1.76E-05 |
| COL15A1 | 4.45795 | 19.47502 | 2.127172 | 1.36E-50 | 3.36E-48 |
| MZB1 | 1.800807 | 13.29111 | 2.883746 | 2.94E-29 | 5.82E-28 |
| CD79B | 0.921329 | 7.508042 | 3.026649 | 3.03E-28 | 5.59E-27 |
| CAMK2A | 0.032265 | 0.184465 | 2.515286 | 6.51E-42 | 3.85E-40 |
| PRRG3 | 0.029971 | 0.155142 | 2.371943 | 5.80E-39 | 2.41E-37 |
| C1S | 17.41641 | 70.27508 | 2.012566 | 1.60E-47 | 2.19E-45 |
| ADGRD1 | 0.102099 | 0.495652 | 2.279352 | 1.11E-33 | 2.87E-32 |
| FGF7 | 0.270165 | 3.008888 | 3.477319 | 1.70E-49 | 3.12E-47 |
| OGN | 0.168619 | 2.224594 | 3.721703 | 3.77E-35 | 1.10E-33 |
| NLRP3 | 0.240912 | 1.019507 | 2.081294 | 8.18E-40 | 3.71E-38 |
| PTCRA | 0.044167 | 0.197568 | 2.161318 | 3.04E-25 | 4.51E-24 |
| SIGLEC8 | 0.096437 | 0.439446 | 2.188032 | 4.23E-27 | 7.15E-26 |
| PLEK | 1.681649 | 9.193759 | 2.450778 | 1.31E-40 | 6.61E-39 |
| CD163 | 1.689972 | 15.7664 | 3.221782 | 1.10E-49 | 2.15E-47 |
| IGLL5 | 3.689327 | 30.65527 | 3.054705 | 2.10E-28 | 3.90E-27 |
| CEACAM4 | 0.146248 | 0.777733 | 2.410855 | 5.69E-35 | 1.62E-33 |
| GATA5 | 0.117083 | 0.882338 | 2.913799 | 1.04E-19 | 1.12E-18 |
| WISP1 | 1.12959 | 5.297073 | 2.229396 | 1.78E-51 | 5.84E-49 |
| ANXA6 | 6.379933 | 27.92846 | 2.130123 | 4.80E-54 | 2.61E-51 |
| ANGPTL7 | 0.044576 | 0.271414 | 2.606151 | 1.37E-13 | 9.64E-13 |
| WNT2 | 0.696161 | 4.54441 | 2.706601 | 4.23E-43 | 2.87E-41 |
| CHI3L1 | 6.217247 | 52.58099 | 3.080193 | 2.45E-39 | 1.06E-37 |
| CD300LB | 0.085647 | 0.417931 | 2.286784 | 3.86E-47 | 4.90E-45 |
| COL1A1 | 90.76566 | 734.6788 | 3.016895 | 1.41E-56 | 1.54E-53 |
| SERPINF1 | 13.04884 | 54.51014 | 2.062603 | 2.45E-46 | 2.70E-44 |
| RARRES2 | 9.412626 | 56.11225 | 2.575647 | 1.19E-44 | 1.02E-42 |
| ISLR | 6.446993 | 54.91354 | 3.090464 | 2.12E-51 | 6.64E-49 |
| ADAMTS12 | 0.467396 | 3.094368 | 2.726927 | 3.07E-53 | 1.45E-50 |
| TNS1 | 2.800381 | 12.14169 | 2.116274 | 2.52E-37 | 8.82E-36 |
| MS4A4A | 1.199259 | 7.690121 | 2.680863 | 3.80E-51 | 1.16E-48 |
| TBX4 | 0.266026 | 1.068313 | 2.005697 | 1.89E-11 | 1.09E-10 |
| MS4A6A | 1.573863 | 8.441267 | 2.42315 | 2.57E-47 | 3.36E-45 |
| C4A | 0.286712 | 1.44055 | 2.328948 | 3.83E-36 | 1.21E-34 |
| PLPP4 | 0.629069 | 4.411608 | 2.810014 | 1.82E-36 | 5.88E-35 |
| CTHRC1 | 10.14088 | 53.13859 | 2.389576 | 1.57E-48 | 2.46E-46 |
| DOK2 | 1.397826 | 6.687086 | 2.258193 | 1.84E-40 | 9.04E-39 |
| PODN | 1.723112 | 10.13026 | 2.555583 | 3.01E-42 | 1.85E-40 |
| KRT85 | 0.017068 | 0.415248 | 4.604593 | 0.005371 | 0.009016 |
| CPA3 | 1.466851 | 7.293861 | 2.313961 | 4.96E-35 | 1.42E-33 |
| SYNDIG1 | 0.375083 | 1.607984 | 2.099969 | 5.57E-40 | 2.59E-38 |
| CXCL12 | 1.624531 | 11.26785 | 2.794117 | 2.25E-45 | 2.12E-43 |
| HCST | 2.654765 | 11.4512 | 2.108843 | 2.90E-34 | 7.78E-33 |
| DIRC1 | 0.028837 | 0.198745 | 2.784939 | 6.93E-39 | 2.85E-37 |
| IL37 | 1.143131 | 0.207072 | -2.46479 | 0.002005 | 0.003646 |
| ATOH8 | 3.72518 | 0.916931 | -2.02243 | 3.13E-06 | 9.06E-06 |
| TIMP2 | 15.34834 | 69.79076 | 2.184953 | 8.17E-50 | 1.67E-47 |
| GGT5 | 1.995208 | 8.349549 | 2.065159 | 4.56E-49 | 7.86E-47 |
| CYTH4 | 0.80144 | 3.815373 | 2.251157 | 4.20E-43 | 2.87E-41 |
| CCL19 | 3.082007 | 23.77483 | 2.947493 | 5.44E-30 | 1.12E-28 |
| SMIM31 | 0.525416 | 0.106339 | -2.30479 | 4.56E-12 | 2.79E-11 |
| ADGRE1 | 0.297587 | 1.196745 | 2.007732 | 2.57E-21 | 3.02E-20 |
| MYO15A | 0.243849 | 0.059483 | -2.03544 | 5.83E-05 | 0.000139 |
| SYNPO2 | 1.149837 | 8.623057 | 2.90677 | 4.14E-23 | 5.37E-22 |
| TREM2 | 3.039837 | 12.97329 | 2.093479 | 2.17E-43 | 1.55E-41 |
| CCL24 | 0.475232 | 2.528831 | 2.411767 | 6.24E-21 | 7.16E-20 |
| ZNF469 | 0.284351 | 1.751074 | 2.622497 | 3.78E-52 | 1.44E-49 |
| TNFSF4 | 0.365212 | 1.608126 | 2.138576 | 3.09E-48 | 4.68E-46 |
| POSTN | 8.224516 | 76.92967 | 3.225538 | 2.37E-55 | 1.97E-52 |
| LY96 | 2.629567 | 11.87292 | 2.174777 | 1.35E-49 | 2.54E-47 |
| MUC2 | 3.160884 | 0.702622 | -2.16951 | 7.66E-09 | 3.19E-08 |
| NNMT | 10.70695 | 57.00392 | 2.412514 | 6.03E-49 | 1.00E-46 |
| PDZRN4 | 0.081544 | 0.573889 | 2.81512 | 1.38E-25 | 2.09E-24 |
| FAM20C | 2.775003 | 12.59261 | 2.182016 | 1.36E-48 | 2.18E-46 |
| LBP | 0.290838 | 3.442665 | 3.565236 | 2.01E-08 | 7.91E-08 |
| ADRB3 | 0.037422 | 0.309679 | 3.048833 | 2.90E-25 | 4.31E-24 |
| CD14 | 12.38185 | 69.60468 | 2.490958 | 1.95E-44 | 1.62E-42 |
| IL10 | 0.190361 | 0.797648 | 2.067014 | 1.68E-41 | 9.29E-40 |
| GFPT2 | 1.335987 | 5.382053 | 2.01025 | 9.34E-49 | 1.51E-46 |
| SIGLEC11 | 0.066062 | 0.279926 | 2.083154 | 1.61E-18 | 1.63E-17 |
| KLHL38 | 0.046169 | 0.21698 | 2.232553 | 8.25E-31 | 1.78E-29 |
| CLC | 0.088688 | 0.739244 | 3.059246 | 1.18E-14 | 9.20E-14 |
| P2RY10 | 0.199106 | 0.94432 | 2.24574 | 1.61E-29 | 3.20E-28 |
| C11orf96 | 5.514276 | 22.09065 | 2.002193 | 3.28E-33 | 8.30E-32 |
| CD33 | 0.127507 | 0.626018 | 2.295625 | 8.83E-46 | 8.72E-44 |
| CLEC10A | 0.533765 | 2.515946 | 2.236825 | 3.08E-28 | 5.68E-27 |
| C3orf80 | 0.322753 | 1.378862 | 2.094974 | 1.05E-46 | 1.25E-44 |
| CD300E | 0.25837 | 1.563143 | 2.596939 | 9.62E-38 | 3.51E-36 |
| FCGR1B | 0.060878 | 0.337803 | 2.472195 | 7.41E-42 | 4.32E-40 |
| RNASE6 | 2.851087 | 14.4383 | 2.340316 | 1.04E-47 | 1.47E-45 |
| LILRA4 | 0.076726 | 0.409277 | 2.415294 | 1.58E-22 | 1.99E-21 |
| DES | 23.92281 | 320.6463 | 3.744524 | 8.78E-29 | 1.67E-27 |
| IL1RL1 | 0.210604 | 0.932246 | 2.146175 | 4.30E-11 | 2.37E-10 |
| PILRA | 0.952311 | 4.242574 | 2.155435 | 5.77E-48 | 8.48E-46 |
| CMKLR1 | 0.572175 | 3.565262 | 2.63948 | 3.39E-44 | 2.62E-42 |
| CD53 | 4.680767 | 23.60724 | 2.334413 | 2.25E-43 | 1.59E-41 |
| TREML1 | 0.145821 | 0.59371 | 2.025558 | 1.71E-21 | 2.04E-20 |
| GPR141 | 0.065418 | 0.263815 | 2.011766 | 6.54E-32 | 1.50E-30 |
| GAS1 | 1.52387 | 8.534628 | 2.485588 | 3.32E-44 | 2.59E-42 |
| C16orf89 | 0.245642 | 1.154913 | 2.233153 | 1.68E-09 | 7.63E-09 |
| NLRP7 | 1.291083 | 8.468953 | 2.713602 | 0.000218 | 0.000472 |
| CADM3 | 0.215589 | 1.969689 | 3.191611 | 7.50E-31 | 1.62E-29 |
| LILRA6 | 0.153124 | 0.719624 | 2.232547 | 1.46E-40 | 7.27E-39 |
| SPIB | 0.924864 | 4.9916 | 2.432189 | 4.76E-20 | 5.16E-19 |
| MMP11 | 8.75525 | 64.27878 | 2.876122 | 6.91E-31 | 1.49E-29 |
| CD19 | 0.324313 | 2.425445 | 2.902789 | 2.13E-18 | 2.13E-17 |
| C1QB | 26.68682 | 167.7179 | 2.651838 | 4.30E-43 | 2.90E-41 |
| ADIPOQ | 0.059437 | 1.086751 | 4.192515 | 3.83E-10 | 1.89E-09 |
| HSPB7 | 0.568575 | 4.943102 | 3.119993 | 5.88E-34 | 1.54E-32 |
| TRAT1 | 0.111351 | 0.507995 | 2.189694 | 2.06E-23 | 2.74E-22 |
| PACRG | 1.16046 | 0.276515 | -2.06927 | 1.14E-12 | 7.41E-12 |
| ZNF831 | 0.047494 | 0.191019 | 2.007908 | 4.76E-26 | 7.46E-25 |
| CTSK | 11.51806 | 90.48683 | 2.973811 | 9.82E-58 | 1.98E-54 |
| ASPN | 1.60583 | 15.77211 | 3.295985 | 4.52E-46 | 4.63E-44 |
| LYVE1 | 0.370169 | 3.560407 | 3.265786 | 1.08E-31 | 2.44E-30 |
| UGT2B15 | 4.841277 | 0.596339 | -3.02118 | 1.48E-05 | 3.85E-05 |
| CYTIP | 1.038695 | 4.243033 | 2.030324 | 4.61E-42 | 2.77E-40 |
| SIGLEC9 | 0.302508 | 1.470762 | 2.281517 | 1.53E-45 | 1.46E-43 |
| XIRP1 | 0.042735 | 0.516821 | 3.596159 | 7.66E-40 | 3.52E-38 |
| FCAR | 0.034029 | 0.227399 | 2.740397 | 1.40E-29 | 2.79E-28 |
| OLFML3 | 4.096806 | 19.11222 | 2.221924 | 2.40E-52 | 9.42E-50 |
| ACTA2 | 30.0246 | 180.6078 | 2.588643 | 2.86E-46 | 3.08E-44 |
| CLLU1OS | 0.062688 | 0.351745 | 2.488282 | 2.22E-07 | 7.57E-07 |
| RSPO3 | 0.211114 | 1.721887 | 3.027894 | 1.34E-50 | 3.36E-48 |
| RGS1 | 4.75289 | 19.9799 | 2.071672 | 1.54E-41 | 8.54E-40 |
| MS4A1 | 0.379082 | 4.730028 | 3.641266 | 8.26E-19 | 8.47E-18 |
| NID2 | 0.87574 | 4.666322 | 2.413712 | 2.02E-44 | 1.66E-42 |
| MCHR1 | 0.062334 | 0.332471 | 2.415143 | 4.82E-30 | 9.96E-29 |
| CHRDL2 | 0.883355 | 7.878222 | 3.156804 | 8.78E-39 | 3.54E-37 |
| IGF1 | 0.077857 | 0.44576 | 2.517364 | 1.49E-36 | 4.85E-35 |
| MSR1 | 0.766434 | 3.8884 | 2.342944 | 7.87E-47 | 9.58E-45 |
| CCL18 | 4.325964 | 34.91554 | 3.012776 | 2.55E-33 | 6.51E-32 |
| CD27 | 1.168142 | 4.749485 | 2.023556 | 1.55E-28 | 2.91E-27 |
| UGT2B28 | 0.410015 | 0.071035 | -2.52908 | 1.41E-13 | 9.89E-13 |
| VWA5B2 | 0.311059 | 0.066847 | -2.21824 | 0.000748 | 0.00147 |
| RUBCNL | 0.167807 | 0.940843 | 2.487151 | 2.89E-44 | 2.30E-42 |
| IL2RA | 0.737717 | 3.547223 | 2.265551 | 2.59E-39 | 1.11E-37 |
| FCGR1A | 0.391355 | 2.30389 | 2.557521 | 2.61E-47 | 3.37E-45 |
| C14orf180 | 0.011215 | 0.255774 | 4.511327 | 1.27E-12 | 8.16E-12 |
| HPSE2 | 0.155831 | 1.564012 | 3.327195 | 2.84E-31 | 6.31E-30 |
| FPR2 | 0.08574 | 0.591626 | 2.786641 | 1.37E-34 | 3.78E-33 |
| GALNT15 | 0.188526 | 1.260119 | 2.740726 | 7.18E-40 | 3.32E-38 |
| GZMK | 0.466236 | 2.897569 | 2.63571 | 4.47E-35 | 1.29E-33 |
| RSPO2 | 0.054916 | 0.375181 | 2.772278 | 6.13E-26 | 9.51E-25 |
| ATP1A2 | 0.111033 | 1.662585 | 3.904371 | 1.23E-12 | 7.93E-12 |
| SPINK4 | 10.08516 | 1.400855 | -2.84785 | 2.36E-07 | 8.04E-07 |
| EPYC | 0.056448 | 1.881024 | 5.05845 | 3.64E-40 | 1.73E-38 |
| CMA1 | 0.102609 | 1.604524 | 3.966923 | 2.74E-20 | 3.02E-19 |
| CHRNA9 | 0.053486 | 0.37876 | 2.82405 | 4.15E-07 | 1.36E-06 |
| SEZ6 | 0.333281 | 0.027103 | -3.62024 | 0.001442 | 0.002697 |
| CCDC80 | 1.562847 | 11.66634 | 2.900103 | 4.17E-54 | 2.35E-51 |
| CYR61 | 26.79497 | 110.5353 | 2.044474 | 2.88E-41 | 1.55E-39 |
| CYTL1 | 0.683266 | 3.201725 | 2.22833 | 9.34E-28 | 1.64E-26 |
| VSTM1 | 0.033604 | 0.186486 | 2.472345 | 5.45E-17 | 5.00E-16 |
| FAM180A | 0.06823 | 0.624956 | 3.195276 | 2.05E-47 | 2.71E-45 |
| FAP | 0.787012 | 3.953921 | 2.328826 | 5.34E-49 | 9.09E-47 |
| MYL9 | 33.71055 | 192.8522 | 2.516224 | 8.22E-44 | 6.01E-42 |
| LGALS12 | 0.051389 | 0.554759 | 3.432332 | 3.39E-22 | 4.21E-21 |
| RNASE2 | 0.385329 | 1.971157 | 2.35488 | 2.10E-41 | 1.14E-39 |
| CYP2C9 | 1.282909 | 0.255385 | -2.32867 | 1.10E-06 | 3.38E-06 |
| FCGR3A | 5.649036 | 33.53215 | 2.569469 | 2.79E-44 | 2.24E-42 |
| KLK4 | 0.229754 | 1.26377 | 2.459569 | 1.19E-14 | 9.21E-14 |
| SLIT2 | 0.261105 | 1.20524 | 2.206621 | 2.32E-39 | 1.01E-37 |
| CCL7 | 0.261269 | 1.2357 | 2.241721 | 1.44E-25 | 2.18E-24 |
| CCIN | 0.039721 | 0.169875 | 2.096506 | 8.86E-41 | 4.55E-39 |
| CCL8 | 1.147643 | 5.13984 | 2.163049 | 1.08E-28 | 2.06E-27 |
| ANXA10 | 33.39961 | 5.581034 | -2.58123 | 3.94E-12 | 2.43E-11 |
| ECM2 | 0.404051 | 1.70135 | 2.074069 | 1.51E-52 | 6.09E-50 |
| PCP4 | 2.927993 | 18.59055 | 2.666585 | 3.18E-12 | 1.98E-11 |
| GZMA | 4.167511 | 21.40716 | 2.360836 | 2.61E-21 | 3.06E-20 |
| ITK | 0.180714 | 0.74747 | 2.048305 | 5.67E-32 | 1.31E-30 |
| SORBS1 | 1.239634 | 7.020868 | 2.501735 | 1.17E-22 | 1.48E-21 |
| ENPP1 | 0.389285 | 1.569802 | 2.011683 | 9.42E-24 | 1.27E-22 |
| CXCL9 | 7.976382 | 40.3852 | 2.34002 | 1.00E-24 | 1.44E-23 |
| CTSG | 0.29124 | 3.45661 | 3.569079 | 1.13E-23 | 1.51E-22 |
| LGALS4 | 15.76563 | 3.588912 | -2.13516 | 2.07E-07 | 7.09E-07 |
| CCL4L2 | 1.071831 | 4.39208 | 2.034827 | 3.47E-22 | 4.30E-21 |
| HSPB6 | 2.038876 | 20.94495 | 3.360756 | 4.15E-29 | 8.12E-28 |
| MFAP5 | 1.368329 | 5.656294 | 2.047442 | 2.40E-42 | 1.50E-40 |
| TRARG1 | 0.031003 | 0.295738 | 3.253852 | 1.55E-08 | 6.23E-08 |
| CFP | 0.227574 | 0.979608 | 2.105871 | 2.55E-23 | 3.36E-22 |
| CHIT1 | 0.25518 | 1.579028 | 2.629451 | 1.25E-14 | 9.65E-14 |
| C7 | 0.985771 | 5.885927 | 2.577945 | 1.07E-17 | 1.02E-16 |
| PTGIS | 2.049227 | 10.67252 | 2.380749 | 7.91E-33 | 1.94E-31 |
| GPC6 | 0.470604 | 3.200926 | 2.765903 | 1.39E-54 | 1.00E-51 |
| PRELP | 1.273165 | 6.652796 | 2.385541 | 3.31E-34 | 8.82E-33 |
| OSCAR | 0.801607 | 3.799991 | 2.245029 | 5.31E-44 | 3.97E-42 |
| CPXM2 | 1.031803 | 5.900899 | 2.515767 | 8.21E-43 | 5.32E-41 |
| COLEC12 | 0.418944 | 3.56129 | 3.087572 | 8.18E-51 | 2.26E-48 |
| PODNL1 | 0.803507 | 3.450306 | 2.102341 | 7.47E-39 | 3.05E-37 |
| ITM2A | 1.944333 | 10.93051 | 2.491013 | 3.93E-30 | 8.17E-29 |
| PNOC | 0.128761 | 0.694842 | 2.431985 | 4.01E-24 | 5.58E-23 |
| UTS2 | 0.152934 | 1.104006 | 2.851765 | 1.03E-08 | 4.23E-08 |
| MAEL | 0.152718 | 1.020732 | 2.740658 | 3.07E-05 | 7.62E-05 |
| GPR174 | 0.109569 | 0.451141 | 2.041737 | 1.88E-18 | 1.89E-17 |
| CRTAC1 | 28.98831 | 3.214428 | -3.17284 | 3.41E-05 | 8.41E-05 |
| BGN | 56.3473 | 292.4984 | 2.376011 | 5.30E-51 | 1.56E-48 |
| CYP4F2 | 1.261684 | 0.226049 | -2.48064 | 1.27E-14 | 9.80E-14 |
| CNR2 | 0.034824 | 0.248256 | 2.833685 | 1.51E-13 | 1.06E-12 |
| ADRA2A | 0.495945 | 1.990948 | 2.005203 | 4.95E-28 | 8.95E-27 |
| SPON1 | 2.168362 | 10.27019 | 2.243785 | 1.07E-41 | 6.03E-40 |
| ATP6V0D2 | 0.105864 | 0.502611 | 2.247231 | 1.86E-15 | 1.53E-14 |
| ONECUT2 | 1.017731 | 0.236547 | -2.10516 | 3.03E-07 | 1.01E-06 |
| LILRB2 | 0.549072 | 3.252972 | 2.56669 | 6.06E-45 | 5.48E-43 |
| MRGPRF | 1.428846 | 7.986887 | 2.482783 | 6.31E-42 | 3.74E-40 |
| PTGDS | 4.428626 | 28.37194 | 2.679534 | 5.53E-34 | 1.46E-32 |
| TMEM130 | 0.122226 | 0.603749 | 2.304398 | 2.97E-28 | 5.49E-27 |
| LEAP2 | 3.957012 | 0.902882 | -2.1318 | 2.85E-10 | 1.43E-09 |
| TNFSF8 | 0.161408 | 0.720853 | 2.158996 | 3.75E-41 | 2.00E-39 |
| JPH2 | 0.781783 | 3.267128 | 2.063182 | 3.62E-32 | 8.51E-31 |
| FCRL5 | 0.069395 | 0.74007 | 3.41475 | 2.02E-29 | 4.01E-28 |
| NKX2-1 | 0.778105 | 0.143944 | -2.43445 | 0.000121 | 0.000275 |
| RBFOX3 | 0.061999 | 0.362744 | 2.548636 | 6.23E-08 | 2.30E-07 |
| GLP2R | 0.034523 | 0.262859 | 2.928649 | 2.85E-16 | 2.47E-15 |
| CILP | 0.282188 | 6.252271 | 4.46965 | 1.09E-38 | 4.34E-37 |
| CD180 | 0.217144 | 1.196223 | 2.461763 | 3.19E-40 | 1.52E-38 |
| VCAN | 3.941126 | 16.64111 | 2.078072 | 8.47E-48 | 1.22E-45 |
| KCNA3 | 0.061996 | 0.279991 | 2.175125 | 3.40E-30 | 7.07E-29 |
| TLR8 | 0.127142 | 0.895748 | 2.816658 | 6.61E-44 | 4.88E-42 |
| APOE | 35.73894 | 167.393 | 2.227671 | 3.38E-37 | 1.17E-35 |
| VSIG4 | 2.051623 | 16.43951 | 3.00233 | 2.70E-48 | 4.19E-46 |
| HTRA3 | 5.332284 | 37.68349 | 2.821107 | 5.32E-47 | 6.71E-45 |
| SFRP2 | 7.673832 | 127.1986 | 4.050991 | 6.54E-51 | 1.89E-48 |
| CD52 | 6.455168 | 30.64581 | 2.247163 | 2.22E-33 | 5.69E-32 |
