# Supplementary material for: CXCL12, a potential modulator of tumor immune microenvironment (TIME) of bladder cancer: From a comprehensive analysis of TCGA database
Source: Front Oncol. 2022 Nov 7;12:1031706. doi: 10.3389/fonc.2022.1031706 (PMC9676933; doi:10.3389/fonc.2022.1031706)
Supplement: Supplementary file 3 [file Table_3.docx]

Supplement Table 3: DEGs obtained from the comparison of high Immune Scores and low Immune Scores

| gene | conMean | treatMean | logFC | pValue | FDR |
| --- | --- | --- | --- | --- | --- |
| WIF1 | 4.553597 | 0.375093 | -3.60169 | 0.003066 | 0.005412 |
| KSR2 | 0.503011 | 0.118069 | -2.09096 | 1.69E-19 | 2.56E-18 |
| IL7R | 1.029574 | 6.19948 | 2.5901 | 6.82E-48 | 5.28E-46 |
| ITGAX | 0.872675 | 3.938779 | 2.174232 | 2.58E-48 | 2.11E-46 |
| SCGB2A1 | 1.291516 | 0.205777 | -2.64991 | 9.70E-05 | 0.000227 |
| TESPA1 | 0.169295 | 0.683329 | 2.013043 | 3.06E-40 | 1.45E-38 |
| MARCO | 0.801685 | 9.355439 | 3.544697 | 2.51E-27 | 6.14E-26 |
| C5AR1 | 1.760871 | 7.743828 | 2.136758 | 1.76E-41 | 8.96E-40 |
| SLAMF1 | 0.128072 | 1.183323 | 3.207811 | 4.65E-57 | 1.65E-54 |
| ALOX5AP | 2.732801 | 12.59697 | 2.204625 | 1.85E-49 | 1.72E-47 |
| SIGLEC10 | 0.462947 | 3.784564 | 3.031209 | 2.17E-54 | 4.05E-52 |
| VNN2 | 0.278712 | 1.383299 | 2.311265 | 2.21E-35 | 7.99E-34 |
| CHRNB2 | 0.28697 | 0.034268 | -3.06595 | 0.001933 | 0.003549 |
| SLCO2B1 | 0.978492 | 5.712278 | 2.545435 | 2.87E-47 | 2.07E-45 |
| CXCL11 | 1.95258 | 25.35909 | 3.69905 | 1.58E-36 | 6.13E-35 |
| ZNF683 | 0.19339 | 2.390804 | 3.627914 | 1.86E-39 | 8.47E-38 |
| SAA1 | 4.862893 | 86.40044 | 4.151152 | 2.75E-24 | 5.72E-23 |
| TNIP3 | 0.104122 | 0.606223 | 2.541566 | 1.74E-40 | 8.45E-39 |
| HAVCR2 | 0.892226 | 5.532396 | 2.632423 | 2.67E-58 | 1.35E-55 |
| SAA2 | 0.542553 | 6.51845 | 3.586694 | 3.91E-22 | 7.13E-21 |
| DOCK2 | 0.298792 | 2.030716 | 2.764775 | 1.26E-55 | 2.70E-53 |
| HBQ1 | 0.420222 | 0.100826 | -2.05928 | 0.033374 | 0.047755 |
| FCRL1 | 0.027944 | 0.412454 | 3.88363 | 4.42E-22 | 8.04E-21 |
| RASGRP4 | 0.129063 | 0.54943 | 2.089861 | 3.75E-46 | 2.49E-44 |
| FCER1G | 8.12199 | 52.77798 | 2.700031 | 2.32E-56 | 6.21E-54 |
| KLRC2 | 0.084524 | 0.366442 | 2.116157 | 1.09E-28 | 2.90E-27 |
| TNFRSF13B | 0.033807 | 0.373729 | 3.466606 | 1.49E-27 | 3.71E-26 |
| TIGIT | 0.289913 | 1.732058 | 2.578797 | 9.97E-51 | 1.08E-48 |
| CCL21 | 4.457674 | 24.71594 | 2.471079 | 1.24E-21 | 2.17E-20 |
| PI16 | 0.420409 | 4.180932 | 3.313959 | 7.69E-08 | 3.00E-07 |
| C20orf141 | 0.095949 | 0.441461 | 2.201949 | 1.84E-19 | 2.78E-18 |
| C4B | 0.389121 | 1.596313 | 2.036451 | 1.19E-28 | 3.15E-27 |
| FOLR2 | 2.581182 | 17.87983 | 2.79223 | 6.49E-39 | 2.87E-37 |
| ITGAM | 0.631107 | 3.736626 | 2.565779 | 1.07E-44 | 6.53E-43 |
| CHRM2 | 0.107724 | 0.433459 | 2.008549 | 0.007329 | 0.012028 |
| ARRDC5 | 0.050776 | 0.210187 | 2.049453 | 6.79E-37 | 2.69E-35 |
| PTX3 | 1.233166 | 5.144046 | 2.060536 | 8.55E-14 | 7.51E-13 |
| CCL23 | 0.182145 | 1.117788 | 2.617484 | 2.01E-31 | 6.14E-30 |
| IL21R | 0.135602 | 1.393075 | 3.360821 | 3.64E-58 | 1.72E-55 |
| SIT1 | 0.491585 | 3.474041 | 2.821102 | 1.69E-52 | 2.30E-50 |
| S100B | 1.073723 | 4.445143 | 2.049608 | 1.58E-35 | 5.80E-34 |
| F2RL2 | 0.46098 | 2.569104 | 2.47849 | 1.78E-16 | 2.06E-15 |
| FOXN4 | 0.708781 | 0.048471 | -3.87014 | 2.16E-06 | 6.65E-06 |
| DDC | 0.643102 | 0.08009 | -3.00535 | 0.001612 | 0.003007 |
| SERTM2 | 0.031531 | 0.174722 | 2.470203 | 3.27E-08 | 1.36E-07 |
| RASAL3 | 0.685037 | 3.748876 | 2.452204 | 1.24E-58 | 7.32E-56 |
| CTLA4 | 0.43138 | 2.650797 | 2.619394 | 9.77E-52 | 1.21E-49 |
| FERMT3 | 2.593839 | 13.66023 | 2.396821 | 7.85E-57 | 2.53E-54 |
| GAPT | 0.100368 | 0.499297 | 2.314596 | 1.95E-29 | 5.45E-28 |
| NELL1 | 0.689299 | 0.036018 | -4.25832 | 9.29E-09 | 4.19E-08 |
| LRRC38 | 0.113952 | 1.127584 | 3.306732 | 7.49E-08 | 2.92E-07 |
| BATF2 | 1.410653 | 7.693634 | 2.447302 | 4.36E-37 | 1.75E-35 |
| PLN | 1.640514 | 7.074156 | 2.10841 | 1.11E-14 | 1.08E-13 |
| HLA-DRB1 | 98.35144 | 501.6304 | 2.350607 | 2.98E-48 | 2.41E-46 |
| P2RY12 | 0.048497 | 0.316275 | 2.705203 | 1.02E-28 | 2.72E-27 |
| STAB1 | 2.753989 | 14.00431 | 2.346277 | 6.87E-40 | 3.19E-38 |
| C1orf162 | 1.236505 | 5.910317 | 2.256967 | 1.20E-55 | 2.61E-53 |
| ASB5 | 0.132915 | 0.683404 | 2.362235 | 1.74E-08 | 7.56E-08 |
| PDCD1LG2 | 0.426841 | 3.490801 | 3.031786 | 3.30E-50 | 3.31E-48 |
| STAP1 | 0.131143 | 1.262373 | 3.266924 | 4.77E-38 | 2.02E-36 |
| CLEC17A | 0.03168 | 0.34006 | 3.424127 | 6.91E-18 | 9.09E-17 |
| PDCD1 | 0.253059 | 2.339117 | 3.208416 | 8.09E-54 | 1.35E-51 |
| KLRB1 | 0.496814 | 2.092391 | 2.074373 | 6.03E-40 | 2.81E-38 |
| SLAMF7 | 1.186421 | 5.645775 | 2.250556 | 4.67E-38 | 1.98E-36 |
| LILRB4 | 0.43733 | 4.315225 | 3.302642 | 1.04E-53 | 1.72E-51 |
| SAA4 | 0.070448 | 0.428179 | 2.603581 | 1.89E-09 | 9.44E-09 |
| KRT33A | 4.309815 | 0.436457 | -3.30371 | 2.74E-08 | 1.15E-07 |
| HLA-DRA | 144.2008 | 706.1865 | 2.29197 | 1.44E-50 | 1.52E-48 |
| WDFY4 | 0.145707 | 1.024453 | 2.813706 | 5.11E-51 | 5.66E-49 |
| FMO2 | 0.176942 | 0.725359 | 2.035416 | 7.16E-17 | 8.52E-16 |
| VPREB3 | 0.537576 | 5.741865 | 3.416978 | 3.80E-08 | 1.56E-07 |
| SCRG1 | 0.135463 | 0.596948 | 2.13971 | 8.60E-08 | 3.32E-07 |
| ADAM2 | 0.537819 | 0.058567 | -3.19895 | 0.004044 | 0.006954 |
| PDZK1IP1 | 10.21607 | 47.0262 | 2.202625 | 3.22E-13 | 2.62E-12 |
| VENTX | 0.114792 | 0.517364 | 2.172155 | 5.16E-34 | 1.77E-32 |
| SIGLEC7 | 0.177556 | 1.02044 | 2.522848 | 3.01E-50 | 3.04E-48 |
| CD3E | 1.104772 | 8.965545 | 3.020643 | 1.72E-59 | 1.87E-56 |
| RAMP1 | 4.412829 | 20.23041 | 2.19675 | 6.06E-25 | 1.31E-23 |
| GBP1 | 7.665959 | 36.5967 | 2.255175 | 5.54E-41 | 2.76E-39 |
| BRINP2 | 0.332095 | 0.039339 | -3.07755 | 0.000706 | 0.001405 |
| VIT | 0.085217 | 0.42479 | 2.317544 | 2.38E-07 | 8.52E-07 |
| BCL2A1 | 1.02114 | 7.533235 | 2.88309 | 7.80E-52 | 9.87E-50 |
| POU2AF1 | 0.687495 | 2.754974 | 2.002618 | 1.48E-19 | 2.26E-18 |
| SERPINA9 | 0.018438 | 0.316984 | 4.103639 | 3.75E-10 | 2.09E-09 |
| PLA2G2D | 0.163043 | 2.932152 | 4.168638 | 7.41E-33 | 2.41E-31 |
| DCSTAMP | 0.034796 | 0.331898 | 3.253732 | 2.53E-26 | 5.89E-25 |
| NCR3 | 0.09696 | 0.675898 | 2.801348 | 4.14E-43 | 2.29E-41 |
| AIF1 | 3.898897 | 28.02561 | 2.845608 | 1.00E-59 | 1.29E-56 |
| JAML | 0.368045 | 1.958496 | 2.411793 | 2.72E-52 | 3.64E-50 |
| FCN1 | 0.207427 | 1.183897 | 2.512866 | 1.73E-29 | 4.85E-28 |
| NCF1 | 0.169384 | 1.613283 | 3.251632 | 4.52E-56 | 1.08E-53 |
| CRH | 57.33141 | 10.50888 | -2.44772 | 2.61E-11 | 1.70E-10 |
| ICOS | 0.177657 | 1.259283 | 2.82544 | 1.18E-51 | 1.44E-49 |
| SPI1 | 2.737989 | 17.49788 | 2.675992 | 7.82E-61 | 2.22E-57 |
| CD209 | 0.436737 | 4.5647 | 3.385684 | 3.16E-45 | 1.96E-43 |
| IL10RA | 0.73169 | 4.881303 | 2.737961 | 2.37E-59 | 2.10E-56 |
| NKG7 | 1.789187 | 19.39032 | 3.437961 | 1.43E-56 | 4.23E-54 |
| MEDAG | 1.347738 | 6.18807 | 2.19895 | 8.62E-18 | 1.12E-16 |
| CXCR3 | 0.266886 | 2.482941 | 3.217753 | 3.26E-56 | 7.97E-54 |
| GYPC | 1.733244 | 7.160781 | 2.046642 | 7.47E-35 | 2.63E-33 |
| POU4F3 | 1.16248 | 0.046667 | -4.63867 | 0.004085 | 0.007022 |
| MYH11 | 10.97082 | 50.53026 | 2.203476 | 3.17E-06 | 9.52E-06 |
| NFAM1 | 0.361294 | 2.206219 | 2.610331 | 1.42E-53 | 2.28E-51 |
| GPR34 | 0.646413 | 3.04863 | 2.237632 | 6.82E-42 | 3.54E-40 |
| CYP1A2 | 10.68326 | 2.09037 | -2.35352 | 0.011706 | 0.018476 |
| EOMES | 0.112409 | 0.582934 | 2.374572 | 2.52E-35 | 9.08E-34 |
| BTLA | 0.074514 | 0.368509 | 2.306111 | 7.26E-41 | 3.59E-39 |
| CD22 | 0.187791 | 2.161317 | 3.524711 | 3.57E-24 | 7.38E-23 |
| SLA | 0.570705 | 3.402584 | 2.575815 | 1.29E-58 | 7.32E-56 |
| DPT | 1.88442 | 10.69121 | 2.504233 | 2.93E-21 | 4.98E-20 |
| THEMIS | 0.073089 | 0.437057 | 2.580103 | 1.57E-45 | 1.00E-43 |
| TYROBP | 11.55813 | 74.89113 | 2.695886 | 6.12E-59 | 4.57E-56 |
| CD300C | 0.256894 | 1.733995 | 2.754857 | 1.80E-50 | 1.86E-48 |
| GLYATL2 | 0.126435 | 0.753783 | 2.575754 | 1.98E-06 | 6.15E-06 |
| EVI2B | 1.086937 | 7.028562 | 2.692961 | 3.79E-59 | 2.98E-56 |
| SAA2-SAA4 | 0.146814 | 3.111542 | 4.405573 | 1.49E-22 | 2.79E-21 |
| SYNM | 2.464196 | 11.47562 | 2.219383 | 4.50E-13 | 3.61E-12 |
| HCK | 1.708494 | 8.999873 | 2.397179 | 2.29E-54 | 4.21E-52 |
| GPR84 | 0.278341 | 1.578823 | 2.503922 | 6.90E-42 | 3.57E-40 |
| SLAMF8 | 1.229207 | 8.589694 | 2.804879 | 6.09E-55 | 1.22E-52 |
| CCL25 | 0.025936 | 0.216824 | 3.063497 | 4.20E-14 | 3.82E-13 |
| HLA-DMB | 2.504462 | 12.10996 | 2.273622 | 4.12E-52 | 5.31E-50 |
| ZBP1 | 0.244884 | 1.592777 | 2.701373 | 4.58E-44 | 2.70E-42 |
| GDF5 | 0.04384 | 0.239146 | 2.447579 | 4.80E-09 | 2.26E-08 |
| GRAP2 | 0.104955 | 0.553283 | 2.39824 | 7.17E-53 | 1.03E-50 |
| CCL5 | 9.195811 | 87.36367 | 3.247985 | 4.82E-48 | 3.79E-46 |
| SOX8 | 0.097301 | 0.393029 | 2.014116 | 2.83E-05 | 7.26E-05 |
| LILRB1 | 0.36209 | 1.811529 | 2.322789 | 1.38E-56 | 4.23E-54 |
| CCL4 | 0.898765 | 7.263912 | 3.01473 | 2.00E-53 | 3.04E-51 |
| ABCD2 | 0.035611 | 0.179023 | 2.329741 | 2.88E-39 | 1.29E-37 |
| CXorf21 | 0.198719 | 1.13797 | 2.517658 | 5.74E-54 | 1.00E-51 |
| TNFAIP8L2 | 0.887596 | 5.240745 | 2.561797 | 7.09E-59 | 4.82E-56 |
| C5AR2 | 0.116581 | 0.713287 | 2.613155 | 2.79E-34 | 9.71E-33 |
| BTK | 0.299041 | 2.044601 | 2.773405 | 1.52E-57 | 6.31E-55 |
| TBC1D10C | 0.462116 | 2.952914 | 2.675812 | 3.50E-52 | 4.55E-50 |
| CD28 | 0.157384 | 0.854942 | 2.441533 | 8.67E-48 | 6.64E-46 |
| C3AR1 | 1.15778 | 8.068161 | 2.800878 | 7.98E-55 | 1.57E-52 |
| TNFSF13B | 0.67104 | 4.177881 | 2.6383 | 1.51E-55 | 3.19E-53 |
| SIGLEC1 | 0.474106 | 4.000483 | 3.076893 | 9.41E-52 | 1.18E-49 |
| CLEC4G | 0.131081 | 1.584505 | 3.595506 | 1.04E-20 | 1.72E-19 |
| PTPN22 | 0.225554 | 1.359048 | 2.591052 | 3.10E-56 | 7.69E-54 |
| APOA2 | 86.00248 | 6.740409 | -3.67347 | 0.004518 | 0.007717 |
| C1QA | 21.60962 | 217.2381 | 3.329532 | 3.59E-58 | 1.72E-55 |
| IRS4 | 0.445009 | 0.017319 | -4.68342 | 9.79E-09 | 4.39E-08 |
| ICAM1 | 6.975461 | 28.4479 | 2.027962 | 7.83E-40 | 3.59E-38 |
| MMP9 | 11.22395 | 90.6913 | 3.014383 | 4.36E-28 | 1.11E-26 |
| PENK | 0.049684 | 0.214343 | 2.109078 | 1.56E-09 | 7.92E-09 |
| CD74 | 189.3526 | 898.0782 | 2.245765 | 1.31E-50 | 1.39E-48 |
| CST7 | 1.837992 | 11.27183 | 2.616518 | 1.12E-55 | 2.48E-53 |
| EVI2A | 0.855758 | 4.274692 | 2.320545 | 1.63E-53 | 2.57E-51 |
| AC136428.1 | 0.10237 | 0.72584 | 2.825864 | 1.49E-18 | 2.08E-17 |
| CD2 | 1.410408 | 11.68457 | 3.050419 | 7.48E-59 | 4.82E-56 |
| TBX21 | 0.100922 | 0.758289 | 2.909506 | 1.36E-50 | 1.44E-48 |
| FCRL2 | 0.026721 | 0.346556 | 3.697045 | 7.47E-26 | 1.70E-24 |
| TCL1A | 0.098668 | 2.61847 | 4.730003 | 1.17E-22 | 2.22E-21 |
| ITGB2 | 2.32361 | 17.48991 | 2.912083 | 1.56E-57 | 6.31E-55 |
| IL17REL | 0.03903 | 0.416001 | 3.41392 | 0.000208 | 0.000456 |
| GBP5 | 0.736422 | 9.986051 | 3.761309 | 2.09E-50 | 2.15E-48 |
| LY86 | 1.088875 | 5.59109 | 2.360291 | 1.33E-52 | 1.83E-50 |
| LAX1 | 0.106949 | 0.72521 | 2.761477 | 4.76E-43 | 2.61E-41 |
| FDCSP | 3.454624 | 108.4455 | 4.972298 | 3.43E-15 | 3.53E-14 |
| P2RX1 | 0.486739 | 2.540218 | 2.383731 | 1.52E-24 | 3.23E-23 |
| SELPLG | 1.949822 | 12.57373 | 2.688998 | 2.50E-61 | 1.77E-57 |
| CD84 | 0.248749 | 1.581211 | 2.668267 | 6.40E-53 | 9.26E-51 |
| IFNG | 0.079202 | 1.151205 | 3.861455 | 3.04E-40 | 1.45E-38 |
| LILRB3 | 0.209597 | 0.882654 | 2.074229 | 1.33E-47 | 1.01E-45 |
| PTPN7 | 0.491583 | 2.818137 | 2.519234 | 4.36E-60 | 6.86E-57 |
| CXorf65 | 0.081862 | 0.413798 | 2.337658 | 1.26E-36 | 4.91E-35 |
| CXCL5 | 1.644816 | 8.032346 | 2.287895 | 7.87E-14 | 6.94E-13 |
| CEACAM3 | 0.034276 | 0.16289 | 2.248651 | 3.04E-30 | 8.83E-29 |
| IL6 | 1.761091 | 8.620428 | 2.29129 | 4.07E-23 | 7.96E-22 |
| SPINK14 | 0.229719 | 0.037771 | -2.60453 | 1.30E-09 | 6.67E-09 |
| ANKRD29 | 0.161593 | 0.793186 | 2.295294 | 2.69E-26 | 6.25E-25 |
| MMP3 | 2.319586 | 19.44708 | 3.067614 | 1.49E-13 | 1.26E-12 |
| SOST | 1.177482 | 5.614634 | 2.253487 | 5.97E-10 | 3.22E-09 |
| SPINK13 | 0.040067 | 0.179462 | 2.163205 | 0.032936 | 0.04721 |
| C1QC | 19.86609 | 171.3162 | 3.108282 | 3.39E-57 | 1.26E-54 |
| ASCL5 | 0.558737 | 0.093224 | -2.5834 | 6.31E-11 | 3.92E-10 |
| VMO1 | 1.587726 | 6.926333 | 2.12513 | 1.09E-30 | 3.22E-29 |
| MYO1G | 0.353774 | 2.242397 | 2.66414 | 3.79E-59 | 2.98E-56 |
| FCRL3 | 0.049034 | 0.491087 | 3.324119 | 5.56E-41 | 2.76E-39 |
| MAP4K1 | 0.769132 | 3.60132 | 2.227222 | 8.89E-45 | 5.47E-43 |
| TPSD1 | 0.241229 | 1.166333 | 2.273506 | 1.42E-11 | 9.52E-11 |
| SH2D1A | 0.157656 | 1.469944 | 3.22091 | 1.59E-55 | 3.31E-53 |
| COMP | 9.827237 | 40.82269 | 2.054513 | 1.47E-10 | 8.66E-10 |
| C2 | 2.607311 | 10.58371 | 2.02121 | 4.06E-40 | 1.91E-38 |
| SCIMP | 0.159312 | 1.165975 | 2.871608 | 3.70E-51 | 4.20E-49 |
| TMIGD3 | 0.419405 | 1.795759 | 2.09818 | 2.57E-42 | 1.38E-40 |
| CD300A | 1.1058 | 5.114325 | 2.209453 | 2.23E-52 | 3.01E-50 |
| CRTAM | 0.110883 | 0.683971 | 2.624894 | 4.48E-48 | 3.55E-46 |
| SLA2 | 0.256924 | 1.772626 | 2.786473 | 1.61E-56 | 4.57E-54 |
| IL12RB1 | 0.275838 | 2.061095 | 2.901515 | 4.50E-58 | 2.06E-55 |
| CD48 | 0.629581 | 5.663679 | 3.169276 | 2.23E-58 | 1.17E-55 |
| CR2 | 0.273908 | 4.102649 | 3.90479 | 2.65E-11 | 1.73E-10 |
| ARHGAP9 | 0.518262 | 3.58869 | 2.791703 | 4.13E-61 | 1.95E-57 |
| CLEC4E | 0.156579 | 1.409514 | 3.170233 | 1.40E-49 | 1.30E-47 |
| PIK3AP1 | 0.704532 | 3.846038 | 2.448636 | 3.34E-50 | 3.33E-48 |
| LAPTM5 | 18.56635 | 81.65142 | 2.136788 | 5.79E-51 | 6.31E-49 |
| MYT1 | 1.638169 | 0.354365 | -2.20878 | 7.54E-08 | 2.94E-07 |
| ADGRG5 | 0.126356 | 0.72329 | 2.517084 | 3.64E-42 | 1.95E-40 |
| CLECL1 | 0.075825 | 0.64915 | 3.097814 | 5.22E-41 | 2.61E-39 |
| CXCR1 | 0.095143 | 0.453163 | 2.251865 | 1.07E-10 | 6.42E-10 |
| KLRD1 | 0.055709 | 0.331946 | 2.574958 | 5.76E-44 | 3.36E-42 |
| CIDEC | 0.184984 | 0.826247 | 2.159175 | 1.84E-08 | 7.95E-08 |
| FPR3 | 2.184316 | 9.572607 | 2.13173 | 1.93E-48 | 1.64E-46 |
| GPR183 | 1.877041 | 9.19412 | 2.292251 | 1.69E-48 | 1.47E-46 |
| ACTG2 | 23.54378 | 100.8048 | 2.098146 | 2.90E-10 | 1.64E-09 |
| CD8A | 0.664542 | 5.584225 | 3.070925 | 4.24E-49 | 3.81E-47 |
| SLFN12L | 0.061488 | 0.346824 | 2.495831 | 2.02E-47 | 1.49E-45 |
| ACTC1 | 2.290097 | 12.07076 | 2.398036 | 3.49E-11 | 2.24E-10 |
| LTF | 3.478837 | 51.48474 | 3.887468 | 2.60E-13 | 2.14E-12 |
| FUT7 | 0.063497 | 0.460186 | 2.857449 | 2.00E-48 | 1.69E-46 |
| JCHAIN | 12.82862 | 68.78962 | 2.422825 | 2.03E-27 | 4.99E-26 |
| KRT81 | 2.533069 | 41.39452 | 4.030481 | 5.69E-13 | 4.53E-12 |
| MRC1 | 1.188675 | 7.798237 | 2.713793 | 2.55E-41 | 1.28E-39 |
| CASP5 | 0.039927 | 0.449059 | 3.49146 | 1.34E-41 | 6.85E-40 |
| HMHB1 | 0.050204 | 0.267373 | 2.412982 | 1.10E-11 | 7.46E-11 |
| LAIR1 | 0.637601 | 3.977912 | 2.641285 | 1.72E-56 | 4.78E-54 |
| PPY | 0.083692 | 1.187401 | 3.82657 | 1.17E-05 | 3.21E-05 |
| FCAMR | 0.041511 | 0.578533 | 3.800848 | 2.80E-05 | 7.19E-05 |
| PSTPIP1 | 0.590796 | 2.381268 | 2.010997 | 1.14E-46 | 7.78E-45 |
| CD4 | 3.248432 | 15.03966 | 2.210957 | 3.17E-52 | 4.19E-50 |
| CXCL13 | 3.401363 | 36.81883 | 3.436259 | 6.46E-41 | 3.20E-39 |
| HLA-DQB2 | 2.740569 | 11.10376 | 2.018501 | 4.43E-34 | 1.53E-32 |
| TCAP | 2.86523 | 0.681068 | -2.07278 | 5.90E-07 | 1.98E-06 |
| SAMSN1 | 0.801825 | 4.328339 | 2.432454 | 5.56E-56 | 1.29E-53 |
| ADAMTS19 | 0.324392 | 0.071951 | -2.17264 | 3.46E-11 | 2.22E-10 |
| LY9 | 0.051416 | 0.403187 | 2.971155 | 4.17E-44 | 2.47E-42 |
| FCGR2A | 2.083446 | 10.94228 | 2.39287 | 2.48E-48 | 2.05E-46 |
| PLD4 | 0.119741 | 0.585858 | 2.290629 | 7.44E-29 | 2.00E-27 |
| G0S2 | 9.5805 | 42.06817 | 2.134556 | 7.70E-22 | 1.37E-20 |
| KRT86 | 1.297271 | 6.596035 | 2.346119 | 7.45E-08 | 2.91E-07 |
| CSF2RB | 1.1542 | 5.219141 | 2.17692 | 3.42E-38 | 1.46E-36 |
| CLEC3A | 0.127869 | 0.764666 | 2.580164 | 0.000579 | 0.001167 |
| CR1 | 0.053436 | 0.441252 | 3.045732 | 1.75E-28 | 4.58E-27 |
| CD37 | 1.114523 | 7.909164 | 2.827099 | 7.31E-54 | 1.23E-51 |
| HLA-DRB5 | 33.20163 | 154.5606 | 2.218847 | 7.32E-46 | 4.78E-44 |
| CD80 | 0.087435 | 0.598731 | 2.775633 | 5.17E-51 | 5.67E-49 |
| SASH3 | 1.101559 | 8.710225 | 2.983164 | 9.19E-63 | 1.30E-58 |
| CLEC4A | 0.649911 | 3.161734 | 2.282402 | 1.19E-52 | 1.65E-50 |
| LILRA2 | 0.064479 | 0.296881 | 2.202996 | 1.72E-45 | 1.09E-43 |
| FGF5 | 0.035609 | 0.298786 | 3.068796 | 2.39E-13 | 1.98E-12 |
| CCR4 | 0.186756 | 0.96414 | 2.368088 | 1.64E-35 | 6.01E-34 |
| GZMH | 0.619236 | 7.411151 | 3.581137 | 3.70E-51 | 4.20E-49 |
| ITGAD | 0.037246 | 0.18429 | 2.306826 | 4.62E-14 | 4.19E-13 |
| TNFRSF17 | 0.183664 | 1.613288 | 3.134861 | 7.28E-28 | 1.84E-26 |
| PTPRC | 0.817828 | 6.314316 | 2.948757 | 1.57E-56 | 4.54E-54 |
| ADAMDEC1 | 0.639105 | 4.788761 | 2.905528 | 6.92E-39 | 3.05E-37 |
| NUGGC | 0.07698 | 0.429913 | 2.481483 | 9.32E-26 | 2.12E-24 |
| CD5 | 0.4943 | 2.737344 | 2.469318 | 3.16E-48 | 2.55E-46 |
| MNDA | 0.730413 | 5.280331 | 2.853844 | 4.70E-56 | 1.11E-53 |
| ALB | 5.120718 | 0.877277 | -2.54524 | 0.03329 | 0.047669 |
| CSF1 | 3.412786 | 13.93693 | 2.029891 | 1.57E-45 | 1.00E-43 |
| CASQ2 | 0.779354 | 3.473148 | 2.155894 | 2.13E-07 | 7.68E-07 |
| CLEC4D | 0.027778 | 0.204017 | 2.876654 | 2.71E-36 | 1.04E-34 |
| BLK | 0.062173 | 0.97501 | 3.971057 | 4.44E-22 | 8.06E-21 |
| FAM129C | 0.049292 | 0.610455 | 3.630455 | 1.07E-12 | 8.23E-12 |
| RIMS4 | 0.491595 | 0.051924 | -3.243 | 0.007679 | 0.01256 |
| HK3 | 0.470217 | 3.687142 | 2.971105 | 2.17E-49 | 2.00E-47 |
| LILRB5 | 0.17092 | 1.345301 | 2.976539 | 1.05E-42 | 5.72E-41 |
| FYB1 | 1.683162 | 6.763153 | 2.006522 | 4.71E-42 | 2.48E-40 |
| SIGLEC12 | 0.185671 | 1.219415 | 2.715372 | 4.69E-27 | 1.14E-25 |
| TSPEAR | 0.310228 | 0.072298 | -2.1013 | 0.02061 | 0.030892 |
| CCR8 | 0.10342 | 0.465604 | 2.170593 | 9.18E-32 | 2.83E-30 |
| TFAP2B | 0.43157 | 0.058344 | -2.88694 | 8.08E-08 | 3.14E-07 |
| PSMB9 | 8.063387 | 41.07883 | 2.348937 | 1.75E-43 | 9.91E-42 |
| SP140 | 0.236571 | 1.583888 | 2.743127 | 3.07E-53 | 4.54E-51 |
| CCER2 | 9.783491 | 0.35191 | -4.79707 | 1.06E-05 | 2.93E-05 |
| UBD | 2.12393 | 16.84379 | 2.987409 | 1.83E-37 | 7.53E-36 |
| SLURP1 | 7.862277 | 1.063705 | -2.88585 | 0.015423 | 0.023765 |
| CNN1 | 12.24048 | 57.03301 | 2.220137 | 5.16E-12 | 3.66E-11 |
| CD300LF | 0.338247 | 1.926754 | 2.510024 | 2.74E-53 | 4.09E-51 |
| PPBP | 0.52016 | 2.308094 | 2.149674 | 0.004593 | 0.007833 |
| HOXC12 | 0.340193 | 0.063992 | -2.41039 | 0.00118 | 0.002256 |
| CCR5 | 0.397256 | 2.951373 | 2.893245 | 2.47E-57 | 9.73E-55 |
| IRF4 | 0.173214 | 1.127119 | 2.702016 | 9.99E-44 | 5.75E-42 |
| CD3G | 0.211154 | 1.327497 | 2.652341 | 1.28E-50 | 1.37E-48 |
| AOAH | 0.351755 | 3.403026 | 3.274174 | 9.90E-59 | 6.10E-56 |
| VSIG8 | 1.733699 | 0.296378 | -2.54834 | 8.84E-07 | 2.90E-06 |
| FGL2 | 1.364061 | 8.386175 | 2.620105 | 3.84E-51 | 4.32E-49 |
| HES6 | 12.95029 | 3.167727 | -2.03146 | 6.45E-07 | 2.15E-06 |
| SLAMF6 | 0.239992 | 2.295302 | 3.257624 | 2.94E-56 | 7.57E-54 |
| IL16 | 0.315082 | 1.418432 | 2.170498 | 7.07E-49 | 6.26E-47 |
| WAS | 1.401008 | 8.05248 | 2.522968 | 7.21E-61 | 2.22E-57 |
| WARS | 16.15639 | 85.94774 | 2.411355 | 1.47E-37 | 6.07E-36 |
| RHOH | 0.173884 | 1.390736 | 2.999653 | 4.41E-57 | 1.60E-54 |
| VCAM1 | 1.72435 | 7.585156 | 2.137126 | 2.98E-32 | 9.38E-31 |
| MRGPRX3 | 0.061957 | 0.574803 | 3.21374 | 0.002811 | 0.005001 |
| CCL13 | 1.512514 | 11.29079 | 2.900126 | 1.28E-31 | 3.93E-30 |
| CD6 | 0.446122 | 2.31439 | 2.375122 | 2.37E-46 | 1.60E-44 |
| PYHIN1 | 0.105116 | 0.828582 | 2.978662 | 5.59E-54 | 9.91E-52 |
| IDO1 | 2.505764 | 27.29735 | 3.445439 | 1.68E-35 | 6.12E-34 |
| CRABP1 | 1.859843 | 0.338012 | -2.46004 | 0.004256 | 0.0073 |
| CCL3 | 1.091917 | 6.587142 | 2.59279 | 3.27E-43 | 1.83E-41 |
| P2RY13 | 0.188728 | 1.387031 | 2.877617 | 5.66E-50 | 5.49E-48 |
| IBSP | 0.607459 | 3.139431 | 2.369644 | 0.000327 | 0.000692 |
| LILRA5 | 0.348104 | 2.832122 | 3.024295 | 1.91E-48 | 1.63E-46 |
| SPN | 0.268048 | 1.571656 | 2.551724 | 6.67E-56 | 1.50E-53 |
| ITGAL | 0.632287 | 4.138862 | 2.710583 | 1.37E-54 | 2.62E-52 |
| FPR1 | 0.765387 | 4.640618 | 2.600056 | 4.61E-42 | 2.44E-40 |
| MEFV | 0.103635 | 0.550359 | 2.408858 | 7.29E-37 | 2.88E-35 |
| SYN2 | 1.177587 | 0.29391 | -2.00239 | 1.80E-05 | 4.77E-05 |
| LCN6 | 0.039892 | 0.198905 | 2.317906 | 3.61E-10 | 2.02E-09 |
| NPHS2 | 0.486823 | 0.117699 | -2.0483 | 2.65E-05 | 6.83E-05 |
| PTGFR | 0.195379 | 0.784367 | 2.00525 | 4.79E-17 | 5.79E-16 |
| CSF1R | 2.737694 | 14.94092 | 2.448236 | 2.28E-49 | 2.09E-47 |
| GRM3 | 0.667187 | 0.113386 | -2.55685 | 5.29E-16 | 5.86E-15 |
| CYBB | 1.756591 | 15.67655 | 3.157758 | 4.96E-55 | 1.00E-52 |
| LRRC25 | 0.623523 | 3.890455 | 2.641423 | 2.06E-54 | 3.90E-52 |
| FLT3 | 0.045828 | 0.205959 | 2.168073 | 1.01E-33 | 3.43E-32 |
| SLC10A1 | 1.578421 | 0.104004 | -3.92377 | 4.13E-07 | 1.42E-06 |
| BIN2 | 0.514492 | 2.700087 | 2.391785 | 1.02E-57 | 4.52E-55 |
| FCER2 | 0.066041 | 1.458245 | 4.464725 | 1.53E-18 | 2.14E-17 |
| HAS1 | 0.258464 | 1.904996 | 2.881753 | 7.66E-14 | 6.77E-13 |
| PARVG | 0.451747 | 2.047639 | 2.180374 | 3.67E-54 | 6.67E-52 |
| ANGPT4 | 0.69393 | 0.128751 | -2.43021 | 0.009966 | 0.015976 |
| UBASH3A | 0.111428 | 0.835665 | 2.906815 | 3.11E-53 | 4.55E-51 |
| F13A1 | 1.968258 | 16.87461 | 3.099863 | 1.55E-27 | 3.84E-26 |
| LTA | 0.116209 | 0.706504 | 2.603977 | 7.10E-48 | 5.47E-46 |
| PAX5 | 0.169286 | 0.836113 | 2.304237 | 3.94E-12 | 2.83E-11 |
| NCKAP1L | 0.518364 | 3.840216 | 2.88915 | 1.33E-57 | 5.71E-55 |
| TAGAP | 0.413192 | 2.141939 | 2.374033 | 5.01E-50 | 4.93E-48 |
| HEPACAM2 | 1.948364 | 0.245838 | -2.98648 | 0.000144 | 0.000327 |
| MPEG1 | 1.292046 | 8.804507 | 2.768585 | 4.47E-55 | 9.18E-53 |
| FAM20A | 0.737109 | 3.303149 | 2.163892 | 3.87E-36 | 1.47E-34 |
| CD79A | 1.270997 | 17.24145 | 3.761849 | 2.33E-29 | 6.47E-28 |
| SFTPD | 0.521894 | 2.69157 | 2.366621 | 0.031827 | 0.045764 |
| CALHM6 | 2.482501 | 12.7127 | 2.356404 | 1.07E-36 | 4.21E-35 |
| GABRP | 2.063915 | 12.12416 | 2.554429 | 1.05E-07 | 3.99E-07 |
| KRT84 | 0.133276 | 0.784651 | 2.557638 | 0.032126 | 0.046162 |
| MZB1 | 1.335201 | 13.75672 | 3.365007 | 1.44E-32 | 4.59E-31 |
| CD7 | 0.986157 | 6.990289 | 2.825463 | 4.62E-54 | 8.29E-52 |
| CD79B | 0.771997 | 7.657374 | 3.310182 | 2.26E-28 | 5.87E-27 |
| NFE4 | 0.235464 | 1.011734 | 2.103253 | 0.000215 | 0.00047 |
| C1S | 16.223 | 71.46849 | 2.139267 | 4.60E-43 | 2.54E-41 |
| LIN28A | 0.495589 | 0.004918 | -6.655 | 0.000781 | 0.001544 |
| NCF2 | 1.912962 | 8.306867 | 2.118496 | 9.75E-47 | 6.70E-45 |
| NLRP3 | 0.208017 | 1.052401 | 2.33891 | 1.09E-44 | 6.66E-43 |
| TLR10 | 0.064247 | 0.640853 | 3.318286 | 1.53E-33 | 5.14E-32 |
| PTCRA | 0.03684 | 0.204895 | 2.475556 | 4.83E-32 | 1.51E-30 |
| PRF1 | 1.937817 | 8.37319 | 2.111345 | 1.14E-53 | 1.86E-51 |
| SIGLEC8 | 0.082071 | 0.453812 | 2.467156 | 2.84E-28 | 7.35E-27 |
| PLEK | 1.168088 | 9.70732 | 3.054924 | 5.71E-56 | 1.30E-53 |
| CD163 | 1.772251 | 15.68412 | 3.14565 | 5.52E-47 | 3.91E-45 |
| IGLL5 | 3.244257 | 31.10034 | 3.26097 | 1.58E-29 | 4.43E-28 |
| DOCK10 | 0.361061 | 1.530006 | 2.083222 | 1.02E-44 | 6.26E-43 |
| CEACAM4 | 0.100922 | 0.823058 | 3.027747 | 1.76E-47 | 1.31E-45 |
| LST1 | 1.028302 | 5.943899 | 2.531146 | 2.07E-60 | 3.67E-57 |
| PIK3R5 | 0.297613 | 1.385462 | 2.218857 | 2.00E-53 | 3.04E-51 |
| CHI3L1 | 5.929886 | 52.86835 | 3.156328 | 2.10E-28 | 5.46E-27 |
| CD300LB | 0.097327 | 0.406252 | 2.061468 | 2.20E-36 | 8.51E-35 |
| MYO1F | 0.787303 | 3.48164 | 2.144777 | 1.43E-56 | 4.23E-54 |
| MS4A4A | 1.151011 | 7.738369 | 2.749128 | 9.24E-50 | 8.79E-48 |
| LTB | 2.02103 | 12.30738 | 2.606361 | 5.93E-46 | 3.89E-44 |
| RGS18 | 0.139999 | 0.698658 | 2.319166 | 1.25E-51 | 1.52E-49 |
| MS4A6A | 1.273466 | 8.741663 | 2.779148 | 7.26E-57 | 2.45E-54 |
| CD163L1 | 0.501607 | 2.038553 | 2.022915 | 2.44E-25 | 5.41E-24 |
| C4A | 0.310747 | 1.416515 | 2.188534 | 3.48E-26 | 8.02E-25 |
| DOK2 | 0.996799 | 7.088112 | 2.830026 | 1.91E-59 | 1.94E-56 |
| EBI3 | 0.672386 | 3.393746 | 2.335517 | 5.10E-42 | 2.67E-40 |
| ZC3H12D | 0.098632 | 0.480904 | 2.285622 | 2.70E-48 | 2.20E-46 |
| KRT85 | 0.012635 | 0.419682 | 5.053845 | 9.04E-05 | 0.000212 |
| NNAT | 65.90267 | 7.957518 | -3.04995 | 0.022827 | 0.03391 |
| CXCL12 | 2.229016 | 10.66337 | 2.258184 | 1.90E-19 | 2.86E-18 |
| HCST | 1.757043 | 12.34892 | 2.813164 | 1.72E-59 | 1.87E-56 |
| IL37 | 1.152146 | 0.198057 | -2.54034 | 0.000315 | 0.000668 |
| MROH2A | 3.529852 | 0.574305 | -2.61972 | 5.61E-10 | 3.04E-09 |
| CYTH4 | 0.588299 | 4.028514 | 2.775627 | 2.19E-59 | 2.07E-56 |
| CCL19 | 2.66444 | 24.1924 | 3.182649 | 1.36E-27 | 3.39E-26 |
| CD247 | 0.585031 | 2.435018 | 2.057348 | 1.33E-49 | 1.25E-47 |
| ADGRE1 | 0.240663 | 1.253669 | 2.381071 | 1.69E-35 | 6.17E-34 |
| MYO15A | 0.251061 | 0.052271 | -2.26395 | 2.62E-07 | 9.30E-07 |
| HLA-DPA1 | 11.73513 | 57.73885 | 2.298709 | 7.42E-49 | 6.53E-47 |
| VNN1 | 0.20907 | 1.695537 | 3.019683 | 1.95E-32 | 6.18E-31 |
| SYNPO2 | 1.922836 | 7.850058 | 2.029468 | 2.82E-08 | 1.18E-07 |
| TREM2 | 3.054292 | 12.95884 | 2.085026 | 9.87E-42 | 5.09E-40 |
| CCL24 | 0.484088 | 2.519975 | 2.380068 | 2.38E-20 | 3.82E-19 |
| LY96 | 2.693179 | 11.8093 | 2.132542 | 1.68E-45 | 1.07E-43 |
| IKZF1 | 0.33344 | 1.768174 | 2.406763 | 6.63E-52 | 8.47E-50 |
| NNMT | 13.02033 | 54.69053 | 2.070525 | 5.13E-31 | 1.54E-29 |
| LBP | 0.2899 | 3.443603 | 3.570291 | 8.25E-05 | 0.000195 |
| FASLG | 0.119286 | 0.845296 | 2.825037 | 3.57E-43 | 1.98E-41 |
| LCP2 | 0.816821 | 4.184921 | 2.357109 | 1.48E-60 | 3.49E-57 |
| CD14 | 9.936644 | 72.04989 | 2.858166 | 1.61E-51 | 1.92E-49 |
| FOLR3 | 0.058893 | 0.410741 | 2.802053 | 1.83E-13 | 1.54E-12 |
| HLA-DQB1 | 7.556829 | 44.66323 | 2.563235 | 2.08E-45 | 1.30E-43 |
| SIGLEC11 | 0.054389 | 0.291599 | 2.42261 | 1.20E-31 | 3.70E-30 |
| CLC | 0.120231 | 0.707701 | 2.557333 | 1.43E-10 | 8.42E-10 |
| P2RY10 | 0.125506 | 1.01792 | 3.019792 | 3.79E-50 | 3.75E-48 |
| GNLY | 0.979081 | 7.987513 | 3.028247 | 1.94E-43 | 1.09E-41 |
| ERVW-1 | 0.244543 | 0.022094 | -3.46834 | 8.17E-08 | 3.17E-07 |
| HLA-DPB1 | 21.89588 | 112.4358 | 2.36037 | 1.11E-54 | 2.16E-52 |
| CD33 | 0.119823 | 0.633703 | 2.402903 | 1.03E-52 | 1.45E-50 |
| IL31RA | 0.050846 | 0.31518 | 2.63196 | 2.64E-20 | 4.21E-19 |
| LGALS2 | 0.731099 | 3.247295 | 2.1511 | 1.44E-35 | 5.33E-34 |
| CLEC10A | 0.377708 | 2.672003 | 2.822577 | 1.92E-43 | 1.08E-41 |
| CD300E | 0.23543 | 1.586083 | 2.752101 | 4.66E-36 | 1.77E-34 |
| FCGR1B | 0.050119 | 0.348561 | 2.797973 | 1.90E-47 | 1.41E-45 |
| RNASE6 | 2.379636 | 14.90975 | 2.647443 | 1.77E-56 | 4.81E-54 |
| LILRA4 | 0.064019 | 0.421984 | 2.720607 | 4.99E-27 | 1.21E-25 |
| DES | 53.93133 | 290.6378 | 2.430027 | 4.47E-10 | 2.46E-09 |
| KIF1A | 1.156996 | 0.281148 | -2.04098 | 0.000373 | 0.00078 |
| IRF8 | 0.887634 | 4.193108 | 2.239984 | 6.49E-49 | 5.78E-47 |
| GPR25 | 0.049773 | 0.326414 | 2.713267 | 3.30E-25 | 7.25E-24 |
| TSPAN32 | 0.093827 | 0.395799 | 2.076688 | 2.51E-39 | 1.13E-37 |
| PILRA | 0.885951 | 4.308934 | 2.282033 | 1.42E-47 | 1.06E-45 |
| CMKLR1 | 0.46752 | 3.669917 | 2.972648 | 3.21E-52 | 4.21E-50 |
| CD53 | 3.462764 | 24.82524 | 2.841812 | 4.85E-60 | 6.88E-57 |
| TREML1 | 0.123924 | 0.615607 | 2.312553 | 3.51E-32 | 1.10E-30 |
| GPR141 | 0.057136 | 0.272098 | 2.251664 | 5.36E-39 | 2.38E-37 |
| KLRC1 | 0.094708 | 0.646909 | 2.772009 | 7.34E-29 | 1.98E-27 |
| CADM3 | 0.319723 | 1.865556 | 2.544713 | 4.96E-17 | 6.00E-16 |
| LILRA6 | 0.126282 | 0.746466 | 2.56343 | 7.33E-47 | 5.12E-45 |
| SPIB | 0.45821 | 5.458253 | 3.574358 | 2.08E-27 | 5.11E-26 |
| SNX20 | 0.247317 | 1.727941 | 2.804622 | 1.99E-60 | 3.67E-57 |
| CD19 | 0.246259 | 2.5035 | 3.345697 | 1.10E-20 | 1.81E-19 |
| C1QB | 18.22286 | 176.1819 | 3.273244 | 6.71E-57 | 2.32E-54 |
| TRAT1 | 0.0593 | 0.560046 | 3.239441 | 5.26E-48 | 4.12E-46 |
| IGSF6 | 0.842847 | 3.712731 | 2.139139 | 1.44E-51 | 1.73E-49 |
| ZNF831 | 0.028806 | 0.209707 | 2.863919 | 2.00E-45 | 1.26E-43 |
| LYVE1 | 0.508214 | 3.422363 | 2.751486 | 1.13E-13 | 9.77E-13 |
| UGT2B15 | 4.521226 | 0.91639 | -2.30268 | 1.87E-05 | 4.94E-05 |
| CYTIP | 0.841406 | 4.440322 | 2.399791 | 1.34E-56 | 4.23E-54 |
| SIGLEC9 | 0.268039 | 1.505231 | 2.48947 | 2.75E-51 | 3.22E-49 |
| XIRP1 | 0.052096 | 0.507461 | 3.284055 | 7.57E-29 | 2.03E-27 |
| FCAR | 0.040461 | 0.220967 | 2.449236 | 2.73E-23 | 5.37E-22 |
| RASGRP2 | 0.383345 | 1.553069 | 2.018408 | 1.30E-24 | 2.78E-23 |
| LAT2 | 0.756181 | 3.60719 | 2.254072 | 3.10E-56 | 7.69E-54 |
| SERPINA1 | 4.962547 | 29.08712 | 2.551227 | 5.29E-39 | 2.36E-37 |
| CLLU1OS | 0.055465 | 0.358968 | 2.694212 | 3.59E-13 | 2.91E-12 |
| CD1C | 0.518941 | 2.351786 | 2.180115 | 7.80E-18 | 1.02E-16 |
| C10orf82 | 0.71139 | 0.145579 | -2.28883 | 3.39E-06 | 1.01E-05 |
| IL4I1 | 1.543248 | 7.470565 | 2.275247 | 3.36E-48 | 2.69E-46 |
| RSPO3 | 0.293787 | 1.639215 | 2.480163 | 1.29E-25 | 2.92E-24 |
| RGS1 | 4.090127 | 20.64266 | 2.335411 | 6.51E-47 | 4.57E-45 |
| LYZ | 26.48415 | 117.344 | 2.147543 | 7.76E-44 | 4.49E-42 |
| MS4A1 | 0.183296 | 4.925815 | 4.748114 | 7.48E-23 | 1.44E-21 |
| CHRDL2 | 1.647511 | 7.114067 | 2.110386 | 2.03E-16 | 2.33E-15 |
| IL22RA2 | 0.056407 | 0.328148 | 2.540408 | 6.57E-22 | 1.17E-20 |
| ASIC5 | 0.579279 | 0.123825 | -2.22595 | 0.00022 | 0.000481 |
| CCL18 | 3.847405 | 35.3941 | 3.201551 | 5.73E-36 | 2.15E-34 |
| DNASE1L3 | 0.354102 | 1.580805 | 2.158424 | 1.10E-19 | 1.69E-18 |
| CD27 | 0.732542 | 5.185085 | 2.823385 | 6.56E-50 | 6.32E-48 |
| CD3D | 2.169515 | 12.41919 | 2.517126 | 1.72E-53 | 2.67E-51 |
| VWA5B2 | 0.313018 | 0.064888 | -2.27022 | 1.09E-05 | 3.00E-05 |
| RUBCNL | 0.174244 | 0.934405 | 2.422937 | 3.45E-40 | 1.63E-38 |
| KIR2DL4 | 0.103922 | 0.742787 | 2.837443 | 4.32E-33 | 1.42E-31 |
| C11orf21 | 0.065553 | 0.342684 | 2.386142 | 5.63E-44 | 3.30E-42 |
| IL2RA | 0.497571 | 3.787369 | 2.928223 | 1.55E-53 | 2.47E-51 |
| FCGR1A | 0.323369 | 2.371876 | 2.874774 | 1.13E-51 | 1.40E-49 |
| CXCL10 | 8.735901 | 103.4446 | 3.565758 | 1.62E-40 | 7.87E-39 |
| F2 | 0.612147 | 0.12115 | -2.33709 | 0.024542 | 0.036185 |
| FPR2 | 0.099802 | 0.577564 | 2.532841 | 9.42E-36 | 3.50E-34 |
| DSG1 | 4.962233 | 0.869726 | -2.51236 | 0.020377 | 0.030559 |
| GZMK | 0.242564 | 3.121241 | 3.685685 | 5.11E-50 | 5.00E-48 |
| HLA-F | 9.991451 | 40.02806 | 2.002246 | 1.28E-37 | 5.32E-36 |
| CMA1 | 0.160257 | 1.546875 | 3.270894 | 1.38E-10 | 8.14E-10 |
| SEZ6 | 0.338394 | 0.02199 | -3.94378 | 0.000414 | 0.000857 |
| SLC39A5 | 1.610021 | 0.130496 | -3.62501 | 2.55E-06 | 7.77E-06 |
| CD40LG | 0.091822 | 0.481074 | 2.38934 | 1.24E-34 | 4.35E-33 |
| VSTM1 | 0.031979 | 0.188111 | 2.55638 | 1.79E-19 | 2.71E-18 |
| STRA8 | 0.192323 | 0.774495 | 2.009723 | 0.022485 | 0.033456 |
| LGALS12 | 0.092648 | 0.5135 | 2.470527 | 2.61E-17 | 3.23E-16 |
| IL2RG | 2.845395 | 20.45938 | 2.846062 | 7.38E-59 | 4.82E-56 |
| RNASE2 | 0.385847 | 1.970639 | 2.352562 | 6.00E-38 | 2.52E-36 |
| C1R | 19.80932 | 84.99872 | 2.101262 | 3.75E-46 | 2.49E-44 |
| PLPPR1 | 0.978821 | 0.18385 | -2.41251 | 1.32E-10 | 7.80E-10 |
| CORO1A | 4.546824 | 19.74807 | 2.118781 | 4.90E-47 | 3.52E-45 |
| CYP2C9 | 1.307169 | 0.231126 | -2.4997 | 0.002758 | 0.004918 |
| FCGR3A | 4.398148 | 34.78304 | 2.983416 | 1.63E-50 | 1.70E-48 |
| CCL7 | 0.133255 | 1.363714 | 3.355275 | 1.71E-27 | 4.21E-26 |
| SCML4 | 0.048058 | 0.218326 | 2.183637 | 3.72E-44 | 2.22E-42 |
| CD86 | 1.009974 | 5.232505 | 2.373183 | 7.55E-57 | 2.49E-54 |
| IL32 | 7.713817 | 31.91592 | 2.048759 | 3.18E-46 | 2.14E-44 |
| UGT2B11 | 0.182882 | 0.04524 | -2.01524 | 4.44E-07 | 1.52E-06 |
| CCL8 | 0.653412 | 5.634071 | 3.108112 | 2.27E-37 | 9.23E-36 |
| CXCR6 | 0.534596 | 2.208005 | 2.046224 | 8.43E-46 | 5.48E-44 |
| FABP9 | 0.246046 | 0.033568 | -2.87378 | 9.00E-06 | 2.52E-05 |
| GZMA | 1.637204 | 23.93747 | 3.869964 | 8.14E-53 | 1.15E-50 |
| ITK | 0.114088 | 0.814096 | 2.835047 | 2.39E-53 | 3.60E-51 |
| CXCL9 | 3.101625 | 45.25996 | 3.867139 | 1.20E-44 | 7.27E-43 |
| CTSG | 0.422974 | 3.324876 | 2.974661 | 8.28E-14 | 7.27E-13 |
| CCL4L2 | 0.724222 | 4.739689 | 2.710289 | 5.07E-37 | 2.03E-35 |
| HLA-DQA2 | 5.61843 | 24.0437 | 2.09742 | 9.20E-31 | 2.73E-29 |
| CFP | 0.200531 | 1.006652 | 2.327671 | 1.12E-25 | 2.54E-24 |
| CHIT1 | 0.277892 | 1.556317 | 2.485541 | 1.84E-12 | 1.38E-11 |
| LAG3 | 0.652901 | 5.886132 | 3.172384 | 9.82E-50 | 9.28E-48 |
| SLITRK2 | 0.044484 | 0.201253 | 2.177642 | 6.30E-10 | 3.38E-09 |
| OSCAR | 0.761842 | 3.839756 | 2.333451 | 6.07E-47 | 4.28E-45 |
| HLA-DQA1 | 4.098272 | 24.90576 | 2.603392 | 7.50E-50 | 7.18E-48 |
| CSF3 | 0.995115 | 5.847814 | 2.554962 | 9.92E-14 | 8.64E-13 |
| PNOC | 0.150623 | 0.67298 | 2.159618 | 1.80E-21 | 3.10E-20 |
| UTS2 | 0.088166 | 1.168774 | 3.728622 | 1.69E-16 | 1.96E-15 |
| GNGT2 | 0.312257 | 1.314542 | 2.073755 | 6.12E-54 | 1.06E-51 |
| GPR174 | 0.060896 | 0.499814 | 3.036982 | 6.05E-38 | 2.54E-36 |
| CRTAC1 | 28.60847 | 3.594271 | -2.99267 | 3.67E-06 | 1.09E-05 |
| TMEM150B | 0.299094 | 1.580029 | 2.401279 | 3.56E-51 | 4.10E-49 |
| KRT40 | 0.806262 | 0.163407 | -2.30278 | 0.000606 | 0.001216 |
| GPR65 | 0.170082 | 0.968497 | 2.509519 | 3.01E-57 | 1.15E-54 |
| CELF3 | 0.324126 | 0.015444 | -4.39142 | 1.40E-06 | 4.46E-06 |
| UNC13C | 0.493049 | 0.090772 | -2.4414 | 0.011093 | 0.017591 |
| CYP4F2 | 1.266737 | 0.220996 | -2.51902 | 4.51E-13 | 3.61E-12 |
| CNR2 | 0.023262 | 0.259818 | 3.481429 | 4.94E-21 | 8.33E-20 |
| ATP6V0D2 | 0.109594 | 0.498881 | 2.18653 | 2.46E-08 | 1.04E-07 |
| TNFRSF9 | 0.147385 | 0.85135 | 2.530161 | 3.03E-51 | 3.52E-49 |
| ONECUT2 | 1.02827 | 0.226008 | -2.18577 | 1.58E-08 | 6.87E-08 |
| LILRB2 | 0.399768 | 3.402276 | 3.089265 | 1.69E-58 | 9.19E-56 |
| ICAM3 | 0.162471 | 0.851436 | 2.389714 | 8.71E-47 | 6.02E-45 |
| GPC3 | 40.80529 | 3.899492 | -3.3874 | 2.56E-06 | 7.81E-06 |
| PTGDS | 6.181071 | 26.6195 | 2.106554 | 1.29E-17 | 1.64E-16 |
| SRGN | 20.98022 | 111.6158 | 2.411441 | 4.09E-51 | 4.56E-49 |
| TNFSF8 | 0.153881 | 0.728379 | 2.242872 | 2.07E-42 | 1.12E-40 |
| IL18RAP | 0.112598 | 0.617951 | 2.456312 | 7.78E-47 | 5.40E-45 |
| BAAT | 2.0969 | 0.050712 | -5.36979 | 7.59E-05 | 0.000181 |
| FCRL5 | 0.059687 | 0.749779 | 3.650982 | 3.13E-28 | 8.03E-27 |
| NKX2-1 | 0.784629 | 0.13742 | -2.51342 | 0.000613 | 0.001229 |
| CCL20 | 4.176418 | 19.36906 | 2.213416 | 7.71E-11 | 4.73E-10 |
| OR2I1P | 1.607338 | 13.07268 | 3.023809 | 3.11E-34 | 1.08E-32 |
| GLP2R | 0.057561 | 0.23982 | 2.058784 | 3.47E-07 | 1.21E-06 |
| LCK | 1.14061 | 5.028397 | 2.140293 | 2.89E-44 | 1.73E-42 |
| ZAP70 | 0.497679 | 2.155927 | 2.11502 | 8.88E-48 | 6.77E-46 |
| CILP | 0.908439 | 5.626021 | 2.630653 | 6.83E-14 | 6.07E-13 |
| CD180 | 0.20125 | 1.212117 | 2.590468 | 1.05E-45 | 6.81E-44 |
| LILRA1 | 0.051327 | 0.264258 | 2.364153 | 9.88E-48 | 7.49E-46 |
| KCNA3 | 0.057426 | 0.284561 | 2.308953 | 1.74E-33 | 5.83E-32 |
| TLR8 | 0.09122 | 0.931669 | 3.352391 | 2.14E-51 | 2.53E-49 |
| APOE | 39.86492 | 163.2671 | 2.034042 | 6.50E-28 | 1.65E-26 |
| VSIG4 | 1.693617 | 16.79751 | 3.310068 | 2.45E-47 | 1.79E-45 |
| GPR171 | 0.217607 | 1.390749 | 2.676064 | 2.40E-47 | 1.76E-45 |
| MT2A | 77.1338 | 329.5436 | 2.095034 | 9.63E-29 | 2.57E-27 |
| CD52 | 4.142304 | 32.95867 | 2.992153 | 2.51E-56 | 6.59E-54 |
