# Supplementary material for: CXCL12, a potential modulator of tumor immune microenvironment (TIME) of bladder cancer: From a comprehensive analysis of TCGA database
Source: Front Oncol. 2022 Nov 7;12:1031706. doi: 10.3389/fonc.2022.1031706 (PMC9676933; doi:10.3389/fonc.2022.1031706)
Supplement: Supplementary file 4 [file Table_4.docx]

Supplement Table 4: DEGs shared by Immune Scores and Stromal Scores

| Gene | logFC |
| --- | --- |
| MARCO | 3.095827 |
| C5AR1 | 2.205185 |
| SLAMF1 | 2.810463 |
| SIGLEC10 | 2.761987 |
| SLCO2B1 | 2.530851 |
| HAVCR2 | 2.367011 |
| DOCK2 | 2.566279 |
| FCRL1 | 3.461287 |
| RASGRP4 | 2.069636 |
| FCER1G | 2.498795 |
| TNFRSF13B | 2.982076 |
| CCL21 | 2.38388 |
| PI16 | 3.723869 |
| C20orf141 | 2.538116 |
| C4B | 2.301758 |
| FOLR2 | 2.805293 |
| ITGAM | 2.314866 |
| CHRM2 | 2.603156 |
| CCL23 | 2.628568 |
| IL21R | 2.753494 |
| SIT1 | 2.441721 |
| F2RL2 | 2.294836 |
| SERTM2 | 2.880532 |
| GAPT | 2.177692 |
| PLN | 2.786895 |
| P2RY12 | 2.622688 |
| STAB1 | 2.29173 |
| ASB5 | 3.053849 |
| PDCD1LG2 | 2.530965 |
| STAP1 | 2.987923 |
| CLEC17A | 3.088893 |
| LILRB4 | 3.041802 |
| WDFY4 | 2.679466 |
| FMO2 | 2.360554 |
| VPREB3 | 3.31446 |
| SCRG1 | 2.791164 |
| VENTX | 2.151965 |
| SIGLEC7 | 2.430698 |
| RAMP1 | 2.252929 |
| BCL2A1 | 2.470692 |
| SERPINA9 | 3.844705 |
| PLA2G2D | 3.704502 |
| DCSTAMP | 3.365333 |
| NCR3 | 2.405 |
| AIF1 | 2.582059 |
| FCN1 | 2.52321 |
| NCF1 | 2.928743 |
| SPI1 | 2.45121 |
| CD209 | 3.179635 |
| IL10RA | 2.478751 |
| MEDAG | 2.509499 |
| GYPC | 2.303653 |
| MYH11 | 2.859216 |
| NFAM1 | 2.410925 |
| GPR34 | 2.231621 |
| CD22 | 3.449812 |
| SLA | 2.372126 |
| DPT | 3.303708 |
| TYROBP | 2.52344 |
| CD300C | 2.580334 |
| GLYATL2 | 2.792841 |
| EVI2B | 2.43662 |
| SYNM | 2.644862 |
| GPR84 | 2.269195 |
| SLAMF8 | 2.592141 |
| CCL25 | 2.834185 |
| GDF5 | 2.740867 |
| CCL5 | 2.898852 |
| CXorf21 | 2.322924 |
| TNFAIP8L2 | 2.298575 |
| C5AR2 | 2.693715 |
| BTK | 2.568089 |
| CD28 | 2.26229 |
| C3AR1 | 2.651716 |
| SIGLEC1 | 2.801225 |
| CLEC4G | 3.562941 |
| C1QA | 3.007278 |
| MMP9 | 3.355245 |
| EVI2A | 2.335948 |
| AC136428.1 | 2.932406 |
| FCRL2 | 3.383843 |
| TCL1A | 4.431116 |
| ITGB2 | 2.661592 |
| LY86 | 2.250251 |
| LAX1 | 2.458512 |
| FDCSP | 5.05793 |
| P2RX1 | 2.853778 |
| SELPLG | 2.480047 |
| CD84 | 2.545397 |
| IL6 | 2.571632 |
| C1QC | 2.81884 |
| FCRL3 | 2.915755 |
| TPSD1 | 2.373805 |
| SH2D1A | 2.635491 |
| COMP | 3.022521 |
| SCIMP | 2.623086 |
| CD48 | 2.748273 |
| CR2 | 3.868966 |
| CLEC4E | 2.95651 |
| PIK3AP1 | 2.254462 |
| CLECL1 | 2.791568 |
| CXCR1 | 2.308159 |
| CIDEC | 2.773647 |
| FPR3 | 2.24967 |
| GPR183 | 2.251164 |
| ACTG2 | 2.724965 |
| ACTC1 | 2.984563 |
| KRT81 | 3.798897 |
| MRC1 | 2.8519 |
| CASP5 | 2.802866 |
| HMHB1 | 2.218227 |
| LAIR1 | 2.465328 |
| PPY | 3.802009 |
| FCAMR | 3.761482 |
| CXCL13 | 3.078379 |
| LY9 | 2.64949 |
| FCGR2A | 2.319957 |
| KRT86 | 2.344405 |
| CR1 | 3.173177 |
| CD37 | 2.57032 |
| SASH3 | 2.593518 |
| LILRA2 | 2.191402 |
| FGF5 | 2.671966 |
| CCR4 | 2.20378 |
| GZMH | 2.835438 |
| TNFRSF17 | 2.878804 |
| PTPRC | 2.610946 |
| ADAMDEC1 | 2.691674 |
| NUGGC | 2.378332 |
| MNDA | 2.587455 |
| CASQ2 | 2.845018 |
| CLEC4D | 2.754202 |
| BLK | 3.566232 |
| FAM129C | 3.379773 |
| HK3 | 2.69672 |
| LILRB5 | 2.80395 |
| CNN1 | 2.878294 |
| CD300LF | 2.264045 |
| AOAH | 2.923848 |
| FGL2 | 2.449723 |
| SLAMF6 | 2.752713 |
| IL16 | 2.111952 |
| WAS | 2.280455 |
| RHOH | 2.669043 |
| CCL13 | 2.742858 |
| P2RY13 | 2.520197 |
| IBSP | 2.437507 |
| LILRA5 | 2.789406 |
| FPR1 | 2.726433 |
| MEFV | 2.259528 |
| LCN6 | 2.564393 |
| PTGFR | 2.196956 |
| CSF1R | 2.476679 |
| CYBB | 2.953026 |
| LRRC25 | 2.473482 |
| FCER2 | 4.134643 |
| HAS1 | 3.368425 |
| F13A1 | 3.197844 |
| NCKAP1L | 2.631793 |
| MPEG1 | 2.589811 |
| FAM20A | 2.189057 |
| CD79A | 3.541727 |
| SFTPD | 2.374416 |
| GABRP | 2.346853 |
| MZB1 | 3.124377 |
| CD79B | 3.168416 |
| C1S | 2.075916 |
| NLRP3 | 2.210102 |
| PTCRA | 2.318437 |
| SIGLEC8 | 2.327594 |
| PLEK | 2.752851 |
| CD163 | 3.183716 |
| IGLL5 | 3.157838 |
| CEACAM4 | 2.719301 |
| CHI3L1 | 3.118261 |
| CD300LB | 2.174126 |
| MS4A4A | 2.714996 |
| MS4A6A | 2.601149 |
| C4A | 2.258741 |
| DOK2 | 2.54411 |
| KRT85 | 4.829219 |
| CXCL12 | 2.526151 |
| HCST | 2.461004 |
| CYTH4 | 2.513392 |
| CCL19 | 3.065071 |
| ADGRE1 | 2.194401 |
| SYNPO2 | 2.468119 |
| TREM2 | 2.089252 |
| CCL24 | 2.395918 |
| LY96 | 2.153659 |
| NNMT | 2.241519 |
| LBP | 3.567764 |
| CD14 | 2.674562 |
| SIGLEC11 | 2.252882 |
| CLC | 2.80829 |
| P2RY10 | 2.632766 |
| CD33 | 2.349264 |
| CLEC10A | 2.529701 |
| CD300E | 2.67452 |
| FCGR1B | 2.635084 |
| RNASE6 | 2.49388 |
| LILRA4 | 2.56795 |
| DES | 3.087275 |
| PILRA | 2.218734 |
| CMKLR1 | 2.806064 |
| CD53 | 2.588112 |
| TREML1 | 2.169055 |
| GPR141 | 2.131715 |
| CADM3 | 2.868162 |
| LILRA6 | 2.397988 |
| SPIB | 3.003274 |
| CD19 | 3.124243 |
| C1QB | 2.962541 |
| TRAT1 | 2.714568 |
| ZNF831 | 2.435914 |
| LYVE1 | 3.008636 |
| CYTIP | 2.215058 |
| SIGLEC9 | 2.385494 |
| XIRP1 | 3.440107 |
| FCAR | 2.594817 |
| CLLU1OS | 2.591247 |
| RSPO3 | 2.754029 |
| RGS1 | 2.203542 |
| MS4A1 | 4.19469 |
| CHRDL2 | 2.633595 |
| CCL18 | 3.107164 |
| CD27 | 2.42347 |
| RUBCNL | 2.455044 |
| IL2RA | 2.596887 |
| FCGR1A | 2.716148 |
| FPR2 | 2.659741 |
| GZMK | 3.160698 |
| CMA1 | 3.618908 |
| VSTM1 | 2.514363 |
| LGALS12 | 2.95143 |
| RNASE2 | 2.353721 |
| FCGR3A | 2.776442 |
| CCL7 | 2.798498 |
| CCL8 | 2.63558 |
| GZMA | 3.1154 |
| ITK | 2.441676 |
| CXCL9 | 3.10358 |
| CTSG | 3.27187 |
| CCL4L2 | 2.372558 |
| CFP | 2.216771 |
| CHIT1 | 2.557496 |
| OSCAR | 2.28924 |
| PNOC | 2.295802 |
| UTS2 | 3.290193 |
| GPR174 | 2.539359 |
| CNR2 | 3.157557 |
| ATP6V0D2 | 2.216881 |
| LILRB2 | 2.827977 |
| PTGDS | 2.393044 |
| TNFSF8 | 2.200934 |
| FCRL5 | 3.532866 |
| GLP2R | 2.493717 |
| CILP | 3.550152 |
| CD180 | 2.526116 |
| KCNA3 | 2.242039 |
| TLR8 | 3.084525 |
| APOE | 2.130856 |
| VSIG4 | 3.156199 |
| CD52 | 2.619658 |
| SCGB2A1 | -2.67899 |
| CHRNB2 | -2.7226 |
| FOXN4 | -3.31958 |
| KRT33A | -3.34778 |
| POU4F3 | -4.84559 |
| TCAP | -2.06883 |
| CCER2 | -4.81756 |
| SLURP1 | -3.01835 |
| NPHS2 | -2.2383 |
| HEPACAM2 | -3.20914 |
| IL37 | -2.50256 |
| MYO15A | -2.14969 |
| UGT2B15 | -2.66193 |
| VWA5B2 | -2.24423 |
| SEZ6 | -3.78201 |
| CYP2C9 | -2.41418 |
| CRTAC1 | -3.08275 |
| CYP4F2 | -2.49983 |
| ONECUT2 | -2.14546 |
| NKX2-1 | -2.47394 |
