# Supplementary material for: CXCL12, a potential modulator of tumor immune microenvironment (TIME) of bladder cancer: From a comprehensive analysis of TCGA database
Source: Front Oncol. 2022 Nov 7;12:1031706. doi: 10.3389/fonc.2022.1031706 (PMC9676933; doi:10.3389/fonc.2022.1031706)
Supplement: Supplementary file 5 [file Table_5.docx]

Supplement Table 5: The number of nodes shared by DEGs in PPI network

| Gene | Count |
| --- | --- |
| ITGAM | 13 |
| CXCL12 | 10 |
| CXCL13 | 8 |
| CXCL9 | 8 |
| FCER1G | 8 |
| ITGB2 | 8 |
| BTK | 7 |
| C4A | 7 |
| C4B | 7 |
| CCL21 | 7 |
| CCL25 | 7 |
| CCL5 | 7 |
| CD79A | 7 |
| CD79B | 7 |
| TYROBP | 7 |
| C1QA | 6 |
| C1QB | 6 |
| CCL13 | 6 |
| CCL19 | 6 |
| CD19 | 6 |
| C1QC | 5 |
| C1S | 5 |
| CXCR1 | 5 |
| LILRB2 | 5 |
| PTPRC | 5 |
| CD14 | 4 |
| CD22 | 4 |
| CD53 | 4 |
| CYBB | 4 |
| LY96 | 4 |
| FCGR1A | 3 |
| FCGR3A | 3 |
| IGLL5 | 3 |
| BLK | 2 |
| C3AR1 | 2 |
| CCR4 | 2 |
| CD180 | 2 |
| CD33 | 2 |
| CR1 | 2 |
| CSF1R | 2 |
| FCN1 | 2 |
| FPR1 | 2 |
| FPR2 | 2 |
| LBP | 2 |
| LY86 | 2 |
| MMP9 | 2 |
| SELPLG | 2 |
| SH2D1A | 2 |
| SPI1 | 2 |
| VSIG4 | 2 |
| WAS | 2 |
| ACTG2 | 1 |
| APOE | 1 |
| C5AR1 | 1 |
| CASP5 | 1 |
| CD28 | 1 |
| CD300E | 1 |
| CD300LB | 1 |
| CLEC4E | 1 |
| CR2 | 1 |
| CTSG | 1 |
| CYP2C9 | 1 |
| CYP4F2 | 1 |
| EVI2A | 1 |
| EVI2B | 1 |
| FCAR | 1 |
| FCGR1B | 1 |
| FCGR2A | 1 |
| HCST | 1 |
| IL6 | 1 |
| ITK | 1 |
| LAIR1 | 1 |
| MNDA | 1 |
| MRC1 | 1 |
| MYH11 | 1 |
| NCF1 | 1 |
| NCKAP1L | 1 |
| NLRP3 | 1 |
| PIK3AP1 | 1 |
| RNASE2 | 1 |
| SLAMF1 | 1 |
| SLAMF6 | 1 |
| TREM2 | 1 |
