# Supplementary material for: CXCL12, a potential modulator of tumor immune microenvironment (TIME) of bladder cancer: From a comprehensive analysis of TCGA database
Source: Front Oncol. 2022 Nov 7;12:1031706. doi: 10.3389/fonc.2022.1031706 (PMC9676933; doi:10.3389/fonc.2022.1031706)
Supplement: Supplementary file 6 [file Table_6.docx]

Supplement Table 6: The most significant DEGs regarding the survival of BC patients through Univariate COX regression analysis of 284 DEGs

| gene | KM | HR | HR.95L | HR.95H | pvalue |
| --- | --- | --- | --- | --- | --- |
| MMP9 | 0.021413 | 1.000233 | 1.000057 | 1.000408 | 0.009393 |
| COMP | 0.009215 | 1.001234 | 1.000233 | 1.002235 | 0.015628 |
| F13A1 | 0.019064 | 1.004874 | 1.001473 | 1.008287 | 0.004937 |
| CXCL12 | 0.049651 | 1.012552 | 1.004674 | 1.020492 | 0.001748 |
