# Supplementary material for: CXCL12, a potential modulator of tumor immune microenvironment (TIME) of bladder cancer: From a comprehensive analysis of TCGA database
Source: Front Oncol. 2022 Nov 7;12:1031706. doi: 10.3389/fonc.2022.1031706 (PMC9676933; doi:10.3389/fonc.2022.1031706)
Supplement: Supplementary file 7 [file Table_7.docx]

Supplement Table 7: The difference test and correlation test between the expression of CXCL12 and TICs.

| TICs | Correlation Test (p-value) | Difference Test (p-value) |
| --- | --- | --- |
| Macrophages M1 | >0.05 | >0.05 |
| Macrophages M0 | >0.05 | >0.05 |
| Macrophages M2 | <0.001 | 0.031 |
| B cells naive | <0.001 | <0.001 |
| B cells memory | >0.05 | >0.05 |
| T cells gamma delta | >0.05 | 0.025 |
| T cells CD4 naive | 0.044 | 0.035 |
| T cells CD4 memory resting | >0.05 | >0.05 |
| T cells CD4 memory activated | >0.05 | >0.05 |
| T cells CD8 | >0.05 | >0.05 |
| T cells regulatory (Tregs) | >0.05 | >0.05 |
| T cells follicular helper | <0.001 | 0.009 |
| NK cells resting | 0.012 | >0.05 |
| NK cells activated | >0.05 | >0.05 |
| Mast cells activated | >0.05 | 0.017 |
| Mast cells resting | 0.003 | 0.004 |
| Dendritic cells resting | 0.003 | 0.024 |
| Dendritic cells activated | <0.001 | >0.05 |
| Neutrophils | >0.05 | >0.05 |
| Eosinophils | >0.05 | 0.028 |
| Monocytes | >0.05 | >0.05 |
| Plasma cells | >0.05 | 0.045 |
